# Childhood Energy Intake Is Associated with Nonalcoholic Fatty Liver Disease in Adolescents^1^,^2^,^3^

**DOI:** 10.3945/jn.114.208397

**Published:** 2015-03-18

**Authors:** Emma L Anderson, Laura D Howe, Abigail Fraser, Corrie Macdonald-Wallis, Mark P Callaway, Naveed Sattar, Chris Day, Kate Tilling, Debbie A Lawlor

**Affiliations:** 4 Medical Research Council Integrative Epidemiology Unit and; 5 School of Social and Community Medicine, University of Bristol, Bristol, United Kingdom;; 6 University Hospitals Bristol NHS Foundation Trust, Bristol, United Kingdom;; 7 Institute of Cardiovascular & Medical Sciences, British Heart Foundation Glasgow Cardiovascular Research Centre, Faculty of Medicine, University of Glasgow, Glasgow, United Kingdom; and; 8 Institute of Cellular Medicine, Faculty of Medical Sciences, Newcastle University, Newcastle, United Kingdom

**Keywords:** diet, energy intake, childhood, NAFLD, fatty liver

## Abstract

**Background:** Greater adiposity is an important risk factor for nonalcoholic fatty liver disease (NAFLD). Thus, it is likely that dietary intake is involved in the development of the disease. Prospective studies assessing the relation between childhood dietary intake and risk of NAFLD are lacking.

**Objective:** This study was designed to explore associations between energy, carbohydrate, sugar, starch, protein, monounsaturated fat, polyunsaturated fat, saturated fat, and total fat intake by youth at ages 3, 7, and 13 y and subsequent (mean age: 17.8 y) ultrasound scan (USS)–measured liver fat and stiffness and serum alanine aminotransferase, aspartate aminotransferase, and γ-glutamyltransferase. We assessed whether observed associations were mediated through fat mass at the time of outcome assessment.

**Methods:** Participants were from the Avon Longitudinal Study of Parents and Children. Trajectories of energy and macronutrient intake from ages 3–13 y were obtained with linear-spline multilevel models. Linear and logistic regression models examined whether energy intake and absolute and energy-adjusted macronutrient intake at ages 3, 7, and 13 y were associated with liver outcomes.

**Results:** Energy intake at all ages was positively associated with liver outcomes; for example, the odds of having a USS-measured liver fat per 100 kcal increase in energy intake at age 3 y were 1.79 (95% CI: 1.14, 2.79). Associations between absolute macronutrient intake and liver outcomes were inconsistent and attenuated to the null after adjustment for total energy intake. The majority of associations attenuated to the null after adjustment for fat mass at the time liver outcomes were assessed.

**Conclusion:** Higher childhood and early adolescent energy intake is associated with greater NAFLD risk, and the macronutrients from which energy intake is derived are less important. These associations appear to be mediated, at least in part, by fat mass at the time of outcome assessment.

## Introduction

Nonalcoholic fatty liver disease (NAFLD)^9^ is a common cause of chronic liver disease in children and adolescents (1), and is associated with fibrosis, insulin resistance, and dyslipidemia, independently of total body fat (2). Because greater adiposity is a key risk factor for NAFLD (3), it is likely that dietary intake is involved in its development. Greater overall energy intake may increase total body fatness, elevating the risk of fat infiltration into the liver by increasing FFA influx from either the diet or via adipose tissue to the liver, with a consequent increase in insulin resistance (3, 4). Alternatively, diet may affect the liver independently of body fatness; studies have shown that manipulation of macro- and micronutrient intake affects inflammation, serum lipids, and insulin resistance, independently of weight loss (5–7).

Most existing evidence for an association between dietary intake and NAFLD in children and adolescents comes from cross-sectional studies (8–12). Results from these studies conflict with some studies reporting that children with NAFLD have diets high in refined carbohydrates (such as fructose) and SFAs and low in PUFAs (8–10), and others reporting no differences in energy or macronutrient intake between children with and without NAFLD (9–11, 12). To our knowledge, just one study has examined the prospective association between diet and NAFLD in children and adolescents (13). This study identified “healthy” and “Western” dietary patterns and found that higher Western dietary pattern scores at age 14 y were associated with a greater risk of NAFLD at age 17 y. In the study, diet was assessed with a single FFQ at age 14 y. Studies have found that FFQs measure diet less accurately than food diaries. Moreover, prospectively assessed repeated measures of dietary intake, which have not been used previously, are important for assessing the age at which associations emerge and whether associations of dietary intake with risk of NAFLD are specific to, or stronger at, a particular age. Thus, the primary objective of this study was to determine prospective associations between childhood energy intake and markers of NAFLD [ultrasound scan (USS)–determined liver fat and stiffness and blood-based indicators of liver function, alanine aminotransferase (ALT), aspartate aminotransferase (AST), and γ-glutamyltransferase (GGT)] assessed at mean age 17.8 y, and to determine whether any observed associations were mediated through total body fat at the time liver outcomes were assessed. As a secondary objective, we explored whether different macronutrients and their subgroups may be driving any associations between energy intake and liver outcomes.

## Methods

### Study population

The Avon Longitudinal Study of Parents and Children (ALSPAC) is a prospective birth cohort from southwest England (14, 15). The study website contains details of all data available through a fully searchable data dictionary (http://www.bris.ac.uk/alspac/researchers/data-access/data-dictionary) (16). Ethical approval for the study was obtained from the ALSPAC Law and Ethics Committee and local research ethics committees. A total of 5081 participants attended the 17–18 y follow-up assessment at mean age 17.8 y. Of these, *n* = 3188 (62.7%) had data available for ALT, AST, and/or GGT. A liver USS substudy (*n* = 1887) was undertaken on a subgroup of participants attending the 17–18 y follow-up (**Supplemental Figure 1**) (2). No participants had a known history of jaundice or hepatitis, were taking medication that would indicate hepatic disease, or were taking medication known to influence liver function.

### Liver outcomes

Participants attending the morning clinic were instructed to fast overnight, and those attending after lunch, for a minimum of 6 h.

Details of USS assessment in the ALSPAC have been published previously (2). Briefly, upper abdominal USS was completed by 1 of 4 trained sonographers using a Siemens Acuson S2000 USS system. Echogenicity was assessed and recorded as present, absent, or uncertain (17). Only 2 scans (0.1%) in total were classified as uncertain. Levels of agreement in identifying echogenicity between the 4 sonographers was 98% or greater, both immediately after training and at 6 mo intervals throughout data collection. Acoustic radiation force impulse imaging (acoustic radiation force impulse measured as shear velocity in meters per second) of the right lobe of the liver was used to measure liver stiffness (our indicator of liver fibrosis) with the use of standard protocols (18).

Fasting blood samples were immediately centrifuged and frozen at −80°C. Measurements were assayed within 3–9 mo after samples were taken, with no previous freeze-thaw cycles. All assays were completed in the same laboratory at the University of Glasgow. ALT, GGT, and AST were measured by Roche Cobas automated analyzers (Roche Diagnostics) with the use of enzymatic methods. All inter- and intra-CVs for these blood-based assays were <5%.

### Dietary intake from ages 3–13 y

Three-day food diaries were mailed to parents when study children were aged 3.5, 5, 7, 10, and 13 y. Parents recorded their child’s diet until the child reached age 10 y. At age 10 y, children recorded their own diet with help from their parents. Diaries were requested for 2 weekdays and 1 weekend day. FFQs were mailed to parents when study children were aged 3, 4, 7, and 9 y. Full details of dietary intake assessment have been published previously (19) and are in **Supplemental Methods**.

### Covariables

Potential confounders considered included the following: maternal age at delivery, parity, education, household social class, and prepregnancy BMI; and participant’s age, sex, pubertal status, and physical activity levels. Total fat mass and height at the time of outcome assessment were considered as potential mediators. A full description of all measures is in Supplemental Methods.

### Eligibility criteria

Participants’ alcohol consumption was assessed at mean ages 16.7 y and 17.8 y with the use of the Alcohol Use Disorders Identification Test (20). Thirteen participants who completed the USS examination and 29 participants with blood-based liver data who were classified as hazardous drinkers at both ages were excluded from this study. To be eligible for inclusion in analyses of USS outcomes, participants had to have valid data for USS-measured liver fat or liver stiffness and at least one measure of dietary intake between ages 3 and 13 y of age (*n* = 1786). For analyses of blood-based liver outcomes, participants were eligible for inclusion if they had data for ALT, AST, or GGT and at least one measure of dietary intake between ages 3 and 13 y (*n* = 3059).

### Statistical analysis

#### Modeling dietary intake trajectories.

As described in a previous publication (21), individual trajectories of energy, total carbohydrate, sugar, starch, total fat, monounsaturated fat, poly unsaturated fat, saturated fat and total protein intake from ages 3–13 y were estimated with the use of linear-spline multilevel models fitted in MLwiN v2.28 with the use of the Stata (StataCorp) command “runmlwin” (22, 23). They were estimated for participants with at least one available food diary or FFQ, under a missing-at-random assumption. Full model details are available on request. Trajectories were used to predict the dietary intake of each participant at ages 3, 7, and 13 y. Energy-adjusted macronutrient intake was calculated by regressing predicted macronutrient intake on predicted energy intake at each age and taking the residuals from this regression. When assessing associations between energy-adjusted macronutrient intake and liver outcomes, we further adjusted for energy intake in all regression models (24).

#### Associations between dietary intake and liver outcomes.

Regression analyses were conducted in Stata v12.0. All continuous outcomes were positively skewed; their ln values were used in regression analyses to ensure residuals were normally distributed. Predicted dietary intake at ages 3, 7, and 13 y were related to continuous outcomes with the use of linear regression, and to the binary USS-measured liver fat variable with the use of logistic regression. Coefficients from regression models including logged variables were back-transformed and are interpreted as a percentage change.

Associations were examined in the following models: *1*) unadjusted, *2*) adjusted for potential confounders, and *3*) additionally adjusted for DXA-determined fat mass, height, and height squared at mean age 17.8 y to determine whether this attenuated any observed associations. Likelihood ratio tests were used to check for gender interactions in all regression analyses.

#### Dealing with missing data and additional analyses.

One-third of eligible participants with USS data (594 of 1786), and 27% of eligible participants with blood-based liver outcomes (821 of 3059) had missing data for potential confounders and/or outcomes. Missing data for any one characteristic varied from 0.03–13% (**Supplemental Tables 1** and **2**). To minimize selection bias and increase efficiency, multivariate multiple imputation was used to impute missing data for potential confounders and outcomes for eligible participants. Full details of this procedure are in Supplemental Methods. Details of a series of sensitivity analyses conducted to verify model assumptions, test the robustness of our findings, and explore whether associations between dietary intake at age 13 y and outcomes might have been confounded by physical activity or pubertal stage are also provided in Supplemental Methods.

## Results

The prevalence of USS-measured fatty liver was 2.5% in males and females. An elevated ALT (>40 U/L) was noted in 4.3% of males (63 of 1474) and 1.6% of females (26 of 1585). Variable distributions were similar among imputed and observed datasets (Supplemental Tables 1 and 2).

We present results with sexes combined; sex was adjusted for in all regression models (*P* values for interaction with sex ≥0.1). Results of the confounder-adjusted model (model 2) are presented in **Tables 1–6**, with results from other models provided in **Supplemental Tables 3–6**.

**TABLE 1:** Associations between energy intake at ages 3, 7, and 13 y and USS- and blood-based liver outcomes at mean age 17.8 y in the imputed data^1^

	Value	*P*
USS-measured liver fat		
3 y	1.79 (1.14, 2.79)	0.01
7 y	1.30 (1.06, 1.60)	0.01
13 y	1.12 (0.84, 1.49)	0.45
USS-measured liver stiffness		
3 y	1 (0, 3)	0.15
7 y	1 (0, 1)	0.06
13 y	0 (−1, 1)	0.43
ALT		
3 y	7 (4,10)	<0.01
7 y	3 (2, 5)	<0.01
13 y	4 (2, 5)	<0.01
AST		
3 y	1 (−0, 3)	0.23
7 y	1 (0, 1)	0.09
13 y	1 (0, 2)	0.04
GGT		
3 y	5 (2, 7)	<0.01
7 y	2 (0, 3)	<0.01
13 y	3 (0, 4)	<0.01

1 Values are ORs (95% CIs) for liver fat and % changes (95% CIs) for liver stiffness, ALT, AST, and GGT. *n* = 1786 for USS-measured liver outcomes at all ages. *n* = 3059 for blood-based liver outcomes at all ages. Coefficients are per 100 kcal increase in energy intake at ages 3, 7, and 13 y. Adjusted for sex, age at outcome assessment, maternal prepregnancy BMI, maternal age, social class, maternal education, and parity. ALT, alanine aminotransferase; AST, aspartate aminotransferase; GGT, γ-glutamyltransferase; USS, ultrasound scan.

**TABLE 2:** Associations between absolute and energy-adjusted carbohydrate intake at ages 3, 7, and 13 y and USS- and blood-based liver outcomes at mean age 17.8 y in the imputed data^1^

	Absolute carbohydrate intake		Energy-adjusted carbohydrate intake	
	Value	*P*	Value	*P*
USS-measured liver fat				
3 y	1.38 (1.06, 1.80)	0.02	1.12 (0.68, 1.84)	0.66
7 y	1.21 (1.04, 1.41)	0.01	1.10 (0.8, 1.52)	0.54
13 y	0.96 (0.82, 1.13)	0.63	0.88 (0.66, 1.17)	0.39
USS-measured liver stiffness				
3 y	1 (0, 1)	0.19	1 (−1, 2)	0.52
7 y	1 (0, 1)	0.04	0 (−1, 1)	0.51
13 y	0 (0, 1)	0.26	1 (0, 2)	0.13
ALT				
3 y	3 (2, 5)	<0.01	−1 (−3, 2)	0.62
7 y	2 (0, 3)	<0.01	0 (−2, 2)	0.89
13 y	2 (0, 3)	<0.01	1 (−1, 2)	0.43
AST				
3 y	1 (0, 2)	0.23	1 (−1, 2)	0.49
7 y	1 (0, 1)	0.02	1 (0, 2)	0.13
13 y	1 (0, 1)	<0.01	1 (0, 2)	0.01
GGT				
3 y	3 (2, 4)	<0.01	2 (0, 5)	0.03
7 y	2 (0, 2)	<0.01	2 (0, 3)	0.01
13 y	2 (0, 2)	<0.01	2 (1, 3)	<0.01

1 Values are ORs (95% CIs) for liver fat and % changes (95% CIs) for liver stiffness, ALT, AST, and GGT. *n* = 1786 for USS-measured liver outcomes at all ages. *n* = 3059 for blood-based liver outcomes at all ages. Coefficients are per 100 kcal increase in energy intake at ages 3, 7, and 13 y. Adjusted for sex, age at outcome assessment, maternal prepregnancy BMI, maternal age, social class, maternal education, and parity. ALT, alanine aminotransferase; AST, aspartate aminotransferase; GGT, γ-glutamyltransferase; USS, ultrasound scan.

**TABLE 3:** Associations between absolute and energy-adjusted sugar intake at ages 3, 7, and 13 y and USS- and blood-based liver outcomes at mean age 17.8 y in the imputed data^1^

	Absolute sugar intake		Energy-adjusted sugar intake	
	Value	*P*	Value	*P*
USS-measured liver fat				
3 y	1.44 (1.02, 2.04)	0.04	1.26 (0.80, 1.98)	0.32
7 y	1.23 (0.99, 1.53)	0.06	1.12 (0.85, 1.47)	0.43
13 y	0.96 (0.79, 1.17)	0.67	0.96 (0.77, 1.22)	0.76
USS-measured liver stiffness				
3 y	1 (0, 2)	0.20	1 (−1, 2)	0.28
7 y	0 (0, 1)	0.18	0 (−1, 1)	0.51
13 y	0 (0, 1)	0.70	0 (−1, 1)	0.74
ALT				
3 y	2 (0, 4)	0.06	−1 (−4, 1)	0.26
7 y	1 (0, 2)	0.05	−1 (−2, 0)	0.16
13 y	0 (−1, 1)	0.36	−1 (−2, 0)	0.06
AST				
3 y	0 (−1, 1)	0.74	0 (−2, 1)	0.89
7 y	1 (0, 1)	0.15	0 (−1, 1)	0.53
13 y	1 (0, 1)	0.02	1 (0, 1)	0.17
GGT				
3 y	4 (2, 5)	<0.01	3 (1, 5)	<0.01
7 y	2 (0, 3)	<0.01	2 (1, 3)	<0.01
13 y	2 (0, 2)	<0.01	1 (0, 2)	0.02

1 Values are ORs (95% CIs) for liver fat and % changes (95% CIs) for liver stiffness, ALT, AST, and GGT. *n* = 1786 for USS-measured liver outcomes at all ages. *n* = 3059 for blood-based liver outcomes at all ages. Coefficients are per 100 kcal increase in energy intake at ages 3, 7, and 13 y. Adjusted for sex, age at outcome assessment, maternal prepregnancy BMI, maternal age, social class, maternal education, and parity. ALT, alanine aminotransferase; AST, aspartate aminotransferase; GGT, γ-glutamyltransferase; USS, ultrasound scan.

**TABLE 4:** Associations between absolute and energy-adjusted starch intake at ages 3, 7, and 13 y and USS- and blood-based liver outcomes at mean age 17.8 y in the imputed data^1^

	Absolute starch intake		Energy-adjusted starch intake	
	Value	*P*	Value	*P*
USS-measured liver fat				
3 y	1.24 (0.86, 1.8)	0.25	0.90 (0.59, 1.38)	0.64
7 y	1.14 (0.90, 1.43)	0.28	0.93 (0.66, 1.3)	0.66
13 y	1.06 (0.79, 1.43)	0.70	0.95 (0.68, 1.33)	0.76
USS-measured liver stiffness				
3 y	0 (−1, 2)	0.50	0 (−1, 1)	0.75
7 y	1 (0, 1)	0.13	0 (−1, 1)	0.75
13 y	1 (0, 2)	0.06	1 (0, 2)	0.09
ALT				
3 y	4 (2, 6)	<0.01	1 (−1, 3)	0.37
7 y	3 (2, 4)	<0.01	1 (0, 3)	0.09
13 y	5 (3, 6)	<0.01	3 (1, 5)	<0.01
AST				
3 y	1 (−1, 2)	0.33	1 (−1, 2)	0.43
7 y	1 (0, 1)	0.11	0 (−1, 2)	0.40
13 y	1 (0, 2)	0.03	1 (0, 2)	0.19
GGT				
3 y	1 (−1, 3)	0.24	−1 (−3, 1)	0.17
7 y	1 (0, 2)	0.09	−1 (−2, 1)	0.36
13 y	2 (0, 3)	0.02	0 (−1, 2)	0.55

1 Values are ORs (95% CIs) for liver fat and % changes (95% CIs) for liver stiffness, ALT, AST, and GGT. *n* = 1786 for USS-measured liver outcomes at all ages. *n* = 3059 for blood-based liver outcomes at all ages. Coefficients are per 100 kcal increase in energy intake at ages 3, 7, and 13 y. Adjusted for sex, age at outcome assessment, maternal prepregnancy BMI, maternal age, social class, maternal education, and parity. ALT, alanine aminotransferase; AST, aspartate aminotransferase; GGT, γ-glutamyltransferase; USS, ultrasound scan.

**TABLE 5:** Associations between absolute and energy-adjusted protein intake at ages 3, 7, and 13 y and USS- and blood-based liver outcomes at mean age 17.8 y in the imputed data^1^

	Absolute protein intake		Energy-adjusted protein intake	
	Value	*P*	Value	*P*
USS-measured liver fat				
3 y	2.60 (1.18, 5.73)	0.02	1.48 (0.59, 3.73)	0.41
7 y	1.75 (1.12, 2.75)	0.02	1.39 (0.74, 2.61)	0.31
13 y	1.68 (1.02, 2.77)	0.04	1.79 (0.93, 3.46)	0.08
USS-measured liver stiffness				
3 y	0 (−3, 2)	0.75	−2 (−5, 1)	0.11
7 y	0 (−0, 2)	0.80	−1 (−3, 0)	0.14
13 y	0 (−2, 1)	0.57	−1 (−3, 0)	0.13
ALT				
3 y	9 (4, 13)	<0.01	2 (−3, 6)	0.53
7 y	6 (3, 8)	<0.01	2 (−1, 6)	0.14
13 y	6 (4, 9)	<0.01	3 (0, 7)	0.04
AST				
3 y	1 (−2, 3)	0.60	0 (−3, 3)	0.92
7 y	1 (−0, 2)	0.36	0 (−2, 2)	0.78
13 y	1 (−0, 3)	0.30	0 (−2, 2)	0.75
GGT				
3 y	5 (0, 8)	0.01	0 (−4, 4)	0.93
7 y	3 (0, 5)	<0.01	5 (−2, 12)	0.20
13 y	4 (2, 6)	<0.01	5 (−1, 13)	0.12

1 Values are ORs (95% CIs) for liver fat and % changes (95% CIs) for liver stiffness, ALT, AST, and GGT. *n* = 1786 for USS-measured liver outcomes at all ages. *n* = 3059 for blood-based liver outcomes at all ages. Coefficients are per 100 kcal increase in energy intake at ages 3, 7, and 13 y. Adjusted for sex, age at outcome assessment, maternal prepregnancy BMI, maternal age, social class, maternal education, and parity. ALT, alanine aminotransferase; AST, aspartate aminotransferase; GGT, γ-glutamyltransferase; USS, ultrasound scan.

**TABLE 6:** Associations between absolute and energy-adjusted fat intake at ages 3, 7, and 13 y and USS- and blood-based liver outcomes at mean age 17.8 y in the imputed data^1^

	Absolute fat intake		Energy-adjusted fat intake	
	Value	*P*	Value	*P*
USS-measured liver fat				
3 y	1.05 (0.96, 1.15)	0.25	0.55 (0.15, 1.99)	0.36
7 y	1.02 (0.98, 1.07)	0.34	0.60 (0.27, 1.35)	0.22
13 y	1.01 (0.95, 1.06)	0.82	0.93 (0.43, 1.99)	0.85
USS-measured liver stiffness				
3 y	0 (0, 0)	0.44	0 (−3, 4)	0.85
7 y	0 (0, 0)	0.25	0 (−2, 2)	0.97
13 y	0 (0, 0)	0.99	−1 (−3, 1)	0.29
ALT				
3 y	1 (0, 1)	<0.01	0 (−3, 4)	0.85
7 y	0 (0, 1)	<0.01	0 (−2, 2)	0.97
13 y	0 (0, 1)	<0.01	−1 (−3, 1)	0.29
AST				
3 y	0 (0, 0)	0.90	−1 (−5, 3)	0.54
7 y	0 (0, 0)	0.75	−2 (−5, 1)	0.14
13 y	0 (0, 0)	0.87	−4 (−6, −1)	<0.01
GGT				
3 y	0 (0, 1)	0.14	−6 (−11, −1)	0.03
7 y	0 (0, 0)	0.30	−5 (−8, −2)	<0.01
13 y	0 (0, 0)	0.81	−6 (−1, −4)	<0.01

1 Values are ORs (95% CIs) for liver fat and % changes (95% CIs) for liver stiffness, ALT, AST, and GGT. *n* = 1786 for USS-measured liver outcomes at all ages. *n* = 3059 for blood-based liver outcomes at all ages. Coefficients are per 100 kcal increase in energy intake at ages 3, 7, and 13 y. Adjusted for sex, age at outcome assessment, maternal prepregnancy BMI, maternal age, social class, maternal education, and parity. ALT, alanine aminotransferase; AST, aspartate aminotransferase; GGT, γ-glutamyltransferase; USS, ultrasound scan.

### 

#### Associations between energy intake at ages 3, 7, and 13 y and liver outcomes.

Energy intake at age 3 y was positively associated with USS-measured liver fat, ALT, and GGT. Energy intake at age 7 y was positively associated with all liver outcomes except AST. Energy intake at 13 y was positively associated with ALT, AST, and GGT (Table 1).

#### Associations between absolute and energy-adjusted macronutrient intake at ages 3, 7, and 13 y and liver outcomes.

Associations between absolute and energy-adjusted carbohydrate, sugar, starch, protein, and fat intake at ages 3, 7, and 13 y and USS- (*n* = 1786) and blood- (*n* = 3059) based liver outcomes are presented in Tables 2–6, respectively. Associations between saturated, monounsaturated and polyunsaturated fat and liver outcomes are in the Supplemental Methods, because they were much the same as those for total fat intake. Although we found evidence of several positive associations between absolute macronutrient intake at 3, 7, and 13 y and each of the USS- and blood-based liver outcomes, there were no consistent patterns, with the exception that energy intake and all absolute macronutrient intake except sugar at ages 3, 7, and 13 y were consistently positively associated with ALT.

After adjusting for energy intake, the majority of the observed positive associations between absolute macronutrient intake and outcomes attenuated to the null (including the consistent positive associations between all macronutrient intake at ages 3, 7, and 13 y and ALT). There were a few exceptions: carbohydrate (Table 2), sugar (Table 3), and fat (Table 6) intake at ages 3, 7, and 13 y all remained positively associated with GGT; carbohydrate and fat intake at age 13 y remained positively associated with AST; and starch (Table 4) and protein (Table 5) intake at age 13 y remained positively associated with ALT.

#### Mediation by fat mass at the time of liver outcome assessment.

After additional adjustment for fat mass at the time liver outcomes were assessed, the majority (49 of 54) of associations between energy intake and absolute macronutrient intake and the USS- and blood-based liver outcomes attenuated toward the null, although some (21) positive associations remained. Most associations between energy-adjusted macronutrient intake and the USS- and blood-based liver outcomes also attenuated toward the null, except that associations between energy-adjusted macronutrients and GGT remained largely unchanged (model 3, Supplemental Tables 3–6).

#### Sensitivity analyses.

Results were similar when restricting to plausible reporters of dietary intake (**Supplemental Table 7**) and when restricting to participants with *1*) complete data on all variables (**Supplemental Table 8**), *2*) at least 2 measures of dietary intake from 3–13 y (**Supplemental Table 9**), and *3*) a measure of dietary intake in each linear-spline period (**Supplemental Table 10**). Results were similar if a bivariate multilevel model was used instead of dietary intake residuals from a univariate multilevel model in regression models (**Supplemental Table 11**), except that CIs were substantially wider. This is expected, given that the bivariate models account for the individual-level dietary intake residuals’ being estimated and not known. Results were also similar after additional adjustment for Alcohol Use Disorders Identification Test scores (**Supplemental Table 12**), and pubertal status and physical activity at age 13 y (**Supplemental Table 13**).

## Discussion

In this study we assessed associations between energy intakes and absolute and energy-adjusted total fat, mono- and polyunsaturated fat, saturated fat, total carbohydrate, sugar, starch, and total protein intake at ages 3, 7, and 13 y and measures of liver health in late adolescence. Because we excluded participants with hazardous alcohol consumption in the 12 mo before liver assessment, and results did not change with further adjustment for alcohol consumption, and because no participants had known hepatic disorders or were on medications that would affect liver function, the associations observed are likely to reflect those of childhood diet with adolescent NAFLD.

Energy intake in childhood and early adolescence was consistently positively associated with markers of NAFLD, but was not associated with liver stiffness. Associations between absolute macronutrient intakes and liver outcomes were inconsistent. There was no evidence that a particular macronutrient was most strongly associated with liver outcomes, or that intake at a particular age was most strongly associated with liver outcomes. However, all absolute macronutrient intake, as well as energy intake, at ages 3, 7, and 13 y were consistently positively associated with ALT. After adjusting for energy intake, the majority of the observed positive associations between absolute macronutrient intake and ALT (and other liver outcomes with positive associations) attenuated to the null. These results suggest that higher energy intake in childhood and early adolescence is associated with greater NAFLD risk, and that the relative proportions of macronutrients from which energy intake is derived are less important. We found some evidence to suggest that fat mass at the time of liver outcome assessment in part mediates the association between childhood dietary intake and NAFLD, with most associations between energy and absolute macronutrient intakes and liver outcomes attenuating toward the null upon adjustment for fat mass.

In a previous study with the use of data from the same cohort, we found that the proportion of participants classified as under- or over-reporters of energy intake increased from 15% at age 3 y to 63% at age 13 y (21). This is perhaps due to the fact that measurement error in dietary intake increases strongly with age, as children begin completing food diaries themselves and eating away from home more often. We attempted to account for this potential source of bias when modeling the trajectories of dietary intake by adjusting for a plausibility of reporting ratio, which has been described in detail elsewhere (21). We also checked results after restricting analyses to participants classified as plausible reporters at ages 3, 7, and 13 y, and they were similar to the main results. This provides some support that implausible reporting of dietary intake does not substantially bias our main results.

### 

#### Comparison with findings from other studies.

We found energy intake in childhood and adolescence to be consistently positively associated with markers of NAFLD, which contradicts findings from studies reporting no associations between energy intake and USS-identified NAFLD (9, 10), ALT, and AST (11). We found absolute fat intake to be positively associated with ALT; however, this was not associated with any other liver outcomes and the associations attenuated to the null after adjustment for energy intake. Consistent with this finding, 2 cross-sectional studies found absolute fat intake to be similar in children with absent, mild, and moderate to severe NAFLD (9, 10), 1 study found no difference in percentage fat intake by ALT status (11), and another study reported no significant differences between children with steatosis compared with steatohepatitis for fraction of energy from fat (12). Published results for subgroups of fat intakes are conflicting: one study found that absolute SFA intake proportionally increased with the degree of hepatic steatosis (9); a second found a high percentage of saturated and a low percentage of polyunsaturated fat intake in children with NAFLD (8); a third found an inverse association between saturated fat intake and NAFLD after adjusting for age, gender, and energy intake (10), and a fourth found no association between percentage total fat or saturated fat intake and steatosis grade (12). These studies were all cross-sectional; to our knowledge, ours is the first large prospective study to relate repeatedly assessed fat and fat subcategories in childhood and early adolescence to NAFLD in adolescence. Taken together, these findings do not provide strong evidence for an effect from dietary fat intake in childhood on subsequent NAFLD risk. However, we would suggest that additional large prospective studies would be useful to clarify this.

Similar to our findings, one study reported no difference in percentage carbohydrate intake between children with differing severities of NAFLD (12), and another found no difference in percentage carbohydrate intake by ALT status (11). Hattar et al. (25) found no difference in the consumption of sweetened beverages between those with NAFLD and the obese and lean control groups. However, high carbohydrate intake and, more specifically, high fructose and sucrose intake have been reported cross-sectionally in children with existing NAFLD (8–10). No studies to date have observed associations between protein intake (absolute or percentage) and liver outcomes. Further large prospective studies are required to clarify associations between macronutrient intake and risk of NAFLD.

Whereas energy intake at all ages was positively associated with most markers of NAFLD, this was not the case for liver stiffness. This could indicate that childhood energy intake affects the likelihood of liver fat accumulation but not liver fibrosis, or that our measure of liver stiffness is not variable enough for us to clearly detect associations in this study. Although we are not aware of other studies with the detailed data that we have to explore this, it is necessary to assess these associations in additional large prospective studies to see if they confirm our findings.

#### Strengths and limitations.

Although power for the binary outcome of USS-measured liver fat was limited because of the small number of cases, we looked at a range of related outcomes including ALT. The USS has a reported sensitivity of 85–90% and specificity of 70–85% for detecting liver fat of at least 10% but lower sensitivity and specificity for lower concentrations of fat (9). Thus, our prevalence estimate most likely reflects the prevalence for the more moderate to severe end of the disease spectrum and consequently our results may be underestimated. Importantly, a recent meta-analysis including 49 different studies found the USS to be reliable and accurate for the detection of steatosis compared with liver histology (26). The missing-at-random assumption underlying multilevel models is likely to hold in our example, because results were similar when restricting analyses to participants with at least 2 dietary intake measures and at least 1 measure in each linear-spline period.

FFQs in this study had no portion size information included; hence, the intakes derived from these may be inaccurate (19). However, we have previously shown that multilevel modeling can be used to efficiently and validly combine data from FFQs and food diaries in this cohort (21). In models in which we adjust for potential mediation by fat mass outcome assessment, there is a potential for collider bias (27). Adjusting for a potential mediator can introduce residual confounding between the exposure and the outcomes if there are unadjusted confounders of the mediator-outcome relation. There is evidence that this generally does not result in considerable bias in practice (28). In this study, the only plausible confounder of the fat mass-NAFLD association not already included in our confounder adjusted regression models was physical activity. We conducted a sensitivity analysis in which we additionally adjusted associations for objectively measured physical activity at age 13 y, and results were very similar to those without adjustment. This cohort consisted largely of white Europeans (97%); thus, our results may not necessarily generalize to other ethnicities.

In conclusion, we have shown that higher childhood and early adolescent energy intake is associated with greater NAFLD risk, and that the macronutrients from which the energy is derived are less important. These associations may be mediated through general adiposity. If these findings are replicated in other prospective studies, they highlight the importance of ensuring healthy energy intake in childhood and adolescence for the prevention of NAFLD.

## Supplementary Material

Online Supporting Material

## Footnotes

9 Abbreviations used: ALSPAC, Avon Longitudinal Study of Parents and Children; ALT, alanine aminotransferase; AST, aspartate aminotransferase; GGT, γ-glutamyltransferase; NAFLD, nonalcoholic fatty liver disease; USS, ultrasound scan.
