# Supplementary material for: Childhood Energy Intake Is Associated with Nonalcoholic Fatty Liver Disease in Adolescents
Source: J Nutr. 2015 Mar 18;145(5):983–9. doi: 10.3945/jn.114.208397 (PMC4410498; doi:10.3945/jn.114.208397)
Supplement: Online Supporting Material [file supp_145_5_983__index.html]

Childhood Energy Intake Is Associated with Nonalcoholic Fatty Liver Disease in Adolescents — Online Supporting Material 

# Childhood Energy Intake Is Associated with Nonalcoholic Fatty Liver Disease in Adolescents

## Online Supporting Material

**Files in this Data Supplement:**

- Online Supporting Material - Text, Figure 1, and Tables 1-13
