# Supplementary material for: Childhood Energy Intake Is Associated with Nonalcoholic Fatty Liver Disease in Adolescents
Source: J Nutr. 2015 Mar 18;145(5):983–9. doi: 10.3945/jn.114.208397 (PMC4410498; doi:10.3945/jn.114.208397)
Supplement: Online Supporting Material [file jn.114.208397_nutrition208397SupplementaryData1.pdf]

Online Supporting Material

Supplemental Figure 1: Participant flow through the study

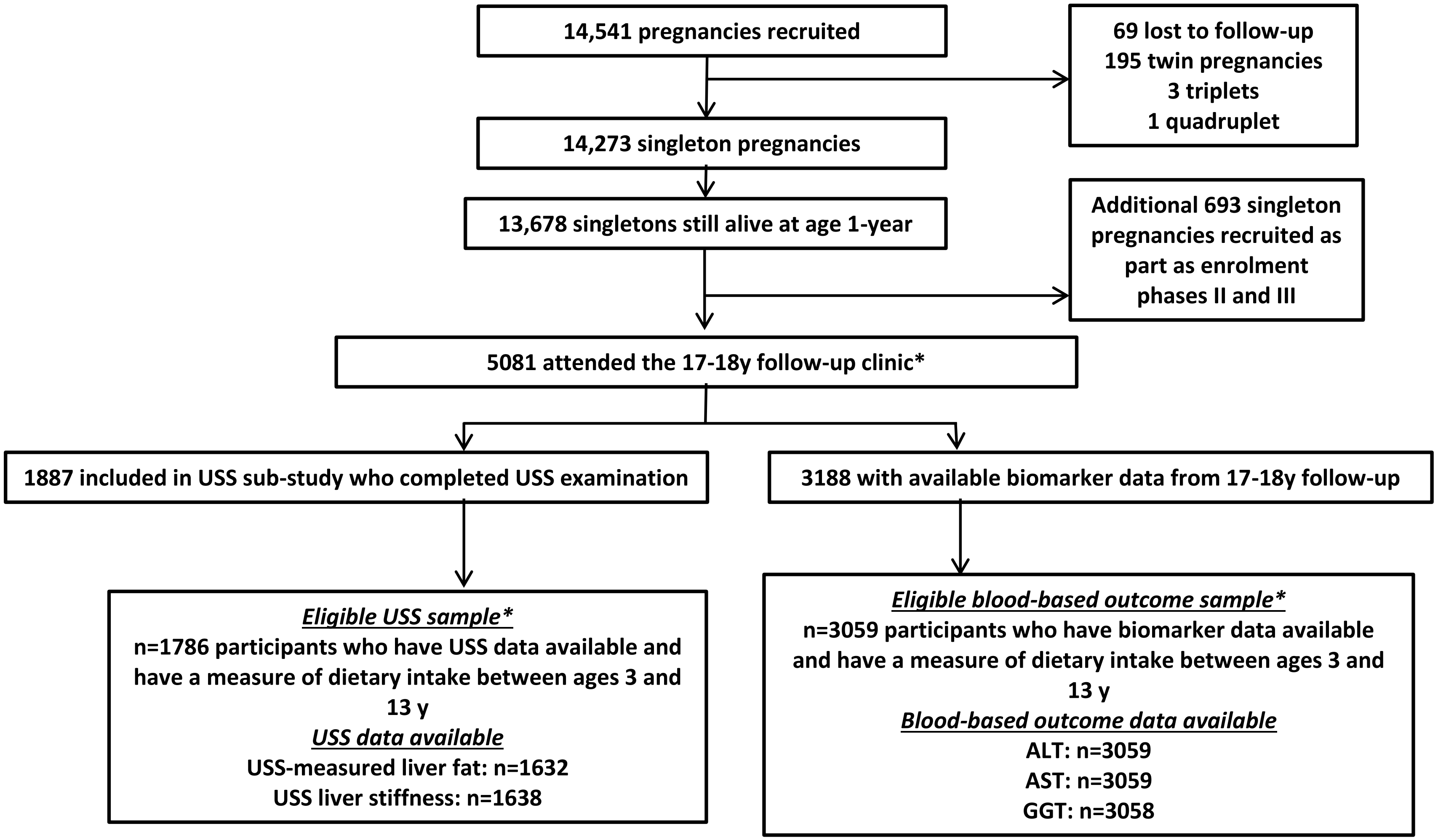

*Participants who had withdrawn, died or were lost to follow-up were not invited to the 17-18y follow-up clinic. Original to this manuscript.

USS – ultrasound scan; ALT- alanine aminotransferase; AST – aspartate aminotransferase; GGT – gamma glutamyl transferase

Online Supporting Material

SUPPLEMENTAL METHODS

Dietary Assessment

Food frequency questionnaires

Mothers and partners were posted self-completion questionnaires at intervals from their enrolment into the study. Once the child was born, parents were asked to fill in parental-report postal questionnaires about the child's health and development. These included a section about their child's diet at age 4 weeks and 6, 15 and 24 months. Each questionnaire contained a range of questions with a section about eating, including the frequency of consumption of specific foods and drinks. As the child grew a full FFQ, adapted from the parental one, was included at ages 3, 4, 7 and 9 y. Each questionnaire was posted to the mother with a reply envelope and if it was not returned within 3 weeks, a postal reminder was sent followed by a second reminder 2 weeks later. A telephone prompt was then given if there was no response after a further month. This has helped with maintaining the response rate to maternal and child questionnaires. The FFQ was used to calculate approximate nutrient intakes by multiplying the weekly frequency of consumption of each food, food group or drink by the nutrient content of a standard portion of that food or drink and summing that for all of the foods and drinks in the questionnaire.(30) The portion sizes and types of foods included in each group were appropriate to the age of the person covered by the questionnaire and for the children no differentiation was made between genders. The original questionnaire covered 43 main food groups with additional questions about the types of bread, milk and fats used and amounts of tea and coffee drunk. As experience was gained in using the FFQ to estimate nutrient intakes, adaptations were made to the questionnaire to enhance the calculations. These included an expansion of the list of food groups to 56 and drink groups to 12, thus providing better assessment of vegetarian foods, milk, soft drinks and alcohol intake. Furthermore, 3-day food diaries collected on the 3.5-year olds gave

insight into foods and drinks consumed in more detail; this information was used to decide the proportion of individual foods to use when estimating the nutrient intake of a food group. For example, 37% (by weight) of fruit eaten was apples and another 37% was bananas with other fruit accounting for 26%, and these proportions were used to calculate the nutrient content of 'fruit' in the FFQ administered at ages 3 and 4 y. Further adaptations were made to the FFQ to accommodate the fact that breaded fish and poultry were eaten more frequently by the children than plain forms. The assessment of what children ate at school was tackled in the FFQ by asking parents to consider in one section 'only food provided by themselves' and in another section, 'food provided by the school'. We suggested that they discussed with the child what had been eaten at school. Additionally, we wrote to schools in the area to obtain copies of menus and serving sizes, so that we could more accurately estimate the nutrient content of the average school meal in the area. When suitable equipment became available, the FFQ was redesigned to allow direct scanning of the data, thus saving time and possible transcription errors. The following number of individuals reported implausible intakes by FFQ and were therefore removed during the data cleaning process: n=329 at age 3 y; n=223 at age 4 y; n=263 at age 7 y and n=233 at age 9 y.

3-day food diaries

A randomly selected subsample of around 10% of the study children, known as Children in Focus, were invited to attend research clinics regularly from age 4 months to 5 y. The child's diet was assessed at 4, 8, 18 months, 3.5 and 5 y. The parents were invited, by post, to record in a structured diary all foods and drinks their child consumed over three individual days (1 only at 4 months): 1 weekend day and 2-week days. They were asked to bring the completed diaries to the clinic where, when funds allowed, they were interviewed briefly by a member of the nutrition team to clarify any anomalies in the diary. Foods and drinks consumed were recorded in household measures on separate pages of the diary to allow specific questions to

be answered. For drinks, details of the amount of concentrate used in diluted drinks, additions such as sugar, total volume offered and amount left over were recorded. For foods, a full description of the food and the amount offered was requested with a separate section for description of leftovers. From 3.5 y onwards a short questionnaire was also included; this asked about the use of vitamin supplements, types of spread normally used on bread, types of milk used and other details of diet to aid dietary coding. The questionnaire was developed in the light of experience gained when interviewing the parent in the previous sweep and proved very successful in giving confidence to the coding of commonly eaten foods. For school meals, parents were instructed to ask the child what they had eaten and to obtain menus, which are often available from schools, to help them. For packed lunches, they were instructed to record everything put into the lunch box and to ask the child to bring back leftover items in the box to be recorded later. The same type of diary was distributed for parental completion for the whole cohort at age 7 y. However, for the data collection at age 10 and 13 y, the diary was redesigned and aimed directly at the child for self-completion with parental help. An accompanying questionnaire was used to elicit help from the parents and gain further information about the foods eaten. The clinic interview, which lasted around 15 minutes, with the child and parent, was vital to obtain additional information and improve the quality of the data obtained. At each age from 18 months onwards, the diet records were coded using the computer programme DIDO (Data In, Diet Out) originally developed by the Dunn Nutrition Unit in Cambridge and shown to improve accuracy and save time compared with other dietary assessment systems.(31) When necessary, the portion sizes used were based on data on average portion sizes for children derived from weighed dietary intakes (32) from two national samples of British children.(33,34) Otherwise portion sizes were based on the manufacturers' information or by adapting adult portion sizes.(35) Nutrient content was calculated for each food and drink consumed and combined to produce average daily nutrient

and food group intakes. The coding of all the records was checked against the original, and the errors were corrected and finally records that produced very high or very low estimates of intake for selected nutrients were rechecked to ensure the quality of the data. No implausible nutrient intakes were reported by food diaries at any measurement occasion.

Assessment of covariables

Maternal age at birth of the child and child's sex were obtained from obstetric records. Questionnaires administered to mothers early in pregnancy asked about parity, educational attainment and pre-pregnancy height and weight. Maternal body mass index (BMI) was calculated as weight in kilograms divided by height in meters squared. Based on questionnaire responses, the highest parental occupation was used to allocate participants to family social class groups using the 1991 British Office of Population and Census Statistics (OPCS) classification. Pubertal stage at the follow-up clinic at mean age 13 y was assessed by a questionnaire in which the respondent was asked to examine line drawings representing the five Tanner stages for pubic hair and to record which drawing most nearly represented the participant's current stage of development.(36,37) Participant's physical activity was objectively measured at mean age 12 y with a uniaxial Actigraph accelerometer (model AM7164 2.2, LLC, Fort Walton Beach, Florida)(38), which was worn for seven days. Actigraphs were downloaded onto a computer and two variables describing levels and patterns of physical activity were derived: average counts per minute (CPM) over the valid measurement period and time spent in moderate to vigorous physical activity (MVPA) in minutes. At the 17-18 y follow-up visit, a lunar prodigy narrow fan beam densitometer was used to perform a whole body dual-energy X-ray absorptiometry (DXA) scan, from which total fat mass was measured. Height was also measured to the nearest 0.1cm using a Harpenden stadiometer with the participant unshod. Age at outcome assessment was calculated from the date of attendance and the participants' birth date.

Online Supporting Material

Multiple imputation procedure

All participants included in this study had trajectories of dietary intake from age 3-13 y, however, one third (n=594/1786) of eligible participants with USS data, and 27% (n=821/3059) of eligible participants with blood-based liver outcomes had missing data for some potential confounders and/or outcomes. To minimise selection bias and increase efficiency, multivariable multiple imputation was used to impute missing data for potential confounders and outcomes, as well as potential predictors of the missing data, for eligible participants. Regression switching was used in Stata, as described by Royston.(39) Twenty cycles of regression switching were carried out and 20 imputation datasets were generated.

We carried out the multivariate multiple imputations separately for males and females and appended the two sets of datasets together to allow us to assess interactions in the multiple imputation datasets. The multiple multivariate imputation approach creates a specified number of copies of the data (in our case, 20 copies) in which missing values are imputed by chained equations, with an appropriate level of randomness. The main results presented in this paper on the multiple imputation datasets are obtained by averaging the results from each of these 20 datasets using Rubin's rules. In this procedure, the standard errors for any regression coefficients (used to calculate p-values and 95% confidence intervals) take account of the uncertainty in the imputations as well as uncertainty in the estimate. The analyses based on these multivariate imputations all include data from the 1786 eligible participants in the USS dataset and the 3059 participants in the blood-based liver outcomes dataset. We also repeated analyses including only those participants with complete data on all variables used in our analyses, i.e. with no missing data (n=1192 in the USS dataset and n=2338 in the blood-based liver outcome dataset). Results from the complete case analyses were very similar to those found based on the multiple imputation datasets (results available upon request).

Online Supporting Material

Additional Analyses

In additional to adjusting for plausibility of reported dietary intake in the modelling of the dietary trajectories, we conducted a sensitivity analyses whereby we restricted analyses to participants classified as plausible reporters at ages 3, 7 and 13 y. To verify the missing at random (MAR) assumption of the multilevel models and to assess potential bias caused by missing data, analyses were repeated after restricting to participants with (i) at least two measures of dietary intake from 3-13 y (n=1734 in the USS dataset and n=2994 in the blood-based liver outcomes dataset) and (ii) at least one measure of dietary intake in each of the age periods defined by our linear-splines (n=1431 in the USS dataset and n=2534 in the blood-based liver outcomes dataset). Analyses were repeated for the continuous liver outcomes using a bivariate multilevel model, whereby two equations are estimated simultaneously: the first dependent variable (equation 1) is dietary intake, described by the linear-spline multilevel model outlined above. The second dependent variable (equation 2) is the continuous liver outcome at age 17.8 y, described by a single individual-level random effect. Regression coefficients are calculated from the covariance matrix of the individual-level random effects, using the Stata command reffadjust. Results of these models were similar to when dietary intake residuals were exported from a univariate multilevel model, and used in subsequent linear regression analyses to assess their associations with the liver outcomes. Analyses were repeated for participants with complete data for all variables included in the analysis (i.e. with no missing data; n=1192 in the USS dataset and n=2338 in the blood-based liver outcome dataset). In additional analyses, we adjusted for AUDIT scores (in addition to removing those with persistent high scores over the year prior to the 17-18y follow-up), to ensure associations were being driven by non-alcoholic, as opposed to alcoholic, fatty liver disease. AUDIT scores from the time of the outcome assessments (mean age 17.8 y) were used if available (80% and 83% of eligible participants in the USS and blood-based liver

outcomes datasets, respectively) and if not, scores assessed in the previous year (mean age 16.7 y) were used. If both were missing, they were imputed as described above. In ALSPAC, participant physical activity data are available at ages 11, 13 and 15 y only. We were therefore unable to adjust for physical activity as a potential confounder in our main confounder adjusted model (which assesses associations with dietary intake at ages 3, 7 and 13 y). As a sensitivity analyses, we assessed associations of dietary intake at age 13 y with the liver outcomes, with additional adjustment for physical activity at age 13 y. We also considered pubertal stage at age 13 y to be a potential confounder of the association between dietary intake at 13 y and NAFLD at mean age 17.8 y. We did not adjust for pubertal stage in our main confounder adjusted models because pubertal stage is not a plausible potential confounder at age 3 y. It is also unlikely to be a potential confounder at age 7 y, as over 95% of participants were pre-pubertal at this age. We therefore conducted a sensitivity analyses in which we assessed associations of dietary intake at age 13 y with the liver outcomes, with additional adjustment for Tanner stage of pubic hair development at mean age 13 y.

Online Supporting Material

Supplemental Table 1: Distribution of variables in the observed (with no missing) and imputation datasets for USS measures

**Imputed Variables**		**Distribution variables in the observed USS dataset (with no missing) n=1192**		**Distribution variables in the imputation dataset for eligible participants with USS data n=1786**		
**Continuous variables**		**Mean ± SE/Median (IQR)**	**Range**	**% Imputed data**	**Mean ± SE/Median (IQR)**	**Range**
USS liver stiffness, m/s (median, IQR)		1.2 (1.1, 1.3)	0.7, 3.7	8	1.2 (1.1, 1.3)	0.6, 4.5
Mean maternal age,y (mean ± SE)		30.0 ± 0.1	17.0, 43.7	3	29.8 ± 0.1	13.7, 43.7
Median maternal BMI, kg/m^2 (median, IQR)		22.2 (20.5, 24.4)	14.2, 44.0	13	22.3 (20.5, 24.7)	12.6, 44.8
Median fat mass at mean age 17.8 y, kg (median, IQR)		16.6 (11.4, 23.0)	1.4, 65.6	4	16.8 (11.4, 23.7)	1.4, 114.9
Mean height at mean age 17.8 y, cm (mean ± SE)		171.1 ± 0.3	146.5, 197.7	3	170.8 ± 0.2	144.6, 201.5
Median age at outcome assessment, months (median, IQR)		214.0 (212.0, 217.0)	200.0, 235.0	0	214.0 (212.0, 217.0)	200.0, 235.0
**Categorical variables**		**Percent**			**Percent**	
USS-measured liver fat	Yes	2.1		9	2.8	
	No	97.9			97.2	
Sex	Male	42.5		0	41.9	
	Female	57.5			58.1	
Parity	0	49.3		8	48.4	
	1	35.9			34.9	
	2+	14.8			16.7	
Head of household social class	Manual	12.0		12	13.8	

	Non-manual	88.0		86.2
Mother's Education	<=O level	51.9	10	53.2
	A level	27.4		27.0
	Degree or above	20.8		19.80
Alcohol consumption in the year prior to outcome assessment[1]	Hazardous	-	9	33.1
	Harmful	-		5.2

[1]In additional analyses on the USS imputed dataset, AUDIT scores were adjusted for. AUDIT scores from the time of the outcome assessments (at mean age 17.8 y) were used if available (80% of eligible participants in the USS dataset). AUDIT scores assessed in the previous year (mean age 16.7 y) were used if the score from the time of the outcome assessments was not available (11% of eligible participants in the USS dataset). The remaining 9% of AUDIT scores for eligible participants in the USS dataset were imputed.

SE- standard error. IQR – interquartile range. USS – ultrasound scan

Online Supporting Material

Supplemental Table 2 : Distribution of variables in the observed (with no missing) and imputation datasets for blood-based liver outcomes

Imputed Variables		Distribution variables in the observed blood-based liver outcomes dataset (with no missing) n=2338		Distribution variables in the imputation dataset for eligible participants with blood-based outcome data n=3059		
**Continuous variables**		**Mean ± SE/Median (IQR)**	**Range**	**% Imputed data**	**Mean ± SE/Median (IQR)**	**Range**
Median ALT, U/L (median, IQR)		15.0 (12.0, 19.5)	2.6, 315.9	0	15.1 (11.9, 19.5)	2.6, 315.9
Median AST, U/L (median, IQR)		19.6 (16.9, 23.2)	7.3, 129.2	0	19.6 (16.8, 23.2)	7.3, 23.2
Median GGT, U/L (median, IQR)		16 (13.0, 21.0)	6.0, 453.0	0.03	16.0 (13.0, 21.0)	6.0, 453.0
Mean maternal age,y (mean ± SE)		30.0 ± 0.1	16.5, 43.3	3.8	29.9 ± 0.1	13.0, 44.1
Median maternal BMI, kg/m^2 (median, IQR)		22.0 (20.5, 24.1)	12.5, 44.0	12.7	22.2 (20.5, 24.3)	12.5, 48.1
Median fat mass at mean age 17.8 y, kg (median, IQR)		15.8 (10.1, 22.0)	1.7, 65.3	0	16.0 (10.3, 22.7)	1.3, 100.9
Mean height at mean age 17.8 y, cm (mean ± SE)		172.4 ± 0.2	146.5, 208.0	0	172.1 ± 0.04	144.6, 208.0
Median age at outcome assessment, months (median, IQR)		213.2 (211.0, 215.2)	195.0, 235.0	0	213.0 (211.0, 215.0)	195.0, 238.0
**Categorical variables**		**Percent**			**Percent**	
Sex	Male	49.5		0	48.2	
	Female	50.5			51.8	
Parity	0	47.86		7.3	47.3	
	1	36.3			35.7	
	2+	15.8			17.0	
Head of household social class	Manual	11.0		10.4	12.5	
	Non-manual	89.0			87.5	

Online Supporting Material

Mother's Education	<=O level	47.2	9.3	48.9
	A level	31.0		30.1
	Degree or above	21.8		21.0
Alcohol consumption in the year prior to outcome assessment[1]	Hazardous	-	6.9	35.0
	Harmful	-		4.8
	Harmful	-		5.2

[1]In additional analyses on the USS imputed dataset, AUDIT scores were adjusted for. AUDIT scores from the time of the outcome assessments (at mean age 17.8 y) were used if available (80% of eligible participants in the USS dataset). AUDIT scores assessed in the previous year (mean age 16.7 y) were used if the score from the time of the outcome assessments was not available (11% of eligible participants in the USS dataset). The remaining 9% of AUDIT scores for eligible participants in the USS dataset were imputed.

SE- standard error. IQR – interquartile range. ALT- alanine aminotransferase; AST – aspartate aminotransferase; GGT – gamma glutamyl transferase.

Online Supporting Material

Supplemental Table 3: Associations of energy intake and absolute macronutrient intakes at ages 3, 7 and 13 y with USS liver outcomes at mean age 17.8 y in the imputed dataset of eligible participants with USS data (N=1786 at all ages)

	Unadjusted			Adjusted for potential confounders[2]			Adjusted for potential confounders mediators[3]		
	USS-measured liver fat								
	OR[1]	95% CI	p	OR[1]	95% CI	p	OR[1]	95% CI	p
Energy intake									
3 y	1.77	(1.23,2.57)	<0.01	1.79	(1.14,2.79)	0.01	1.17	(0.68, 1.99)	0.57
7 y	1.39	(1.15,1.67)	<0.01	1.30	(1.06,1.60)	0.01	1.05	(0.82, 1.36)	0.70
13 y	1.07	(0.91,1.26)	0.38	1.12	(0.84,1.49)	0.45	0.81	(0.58, 1.13)	0.21
Carbohydrate intake									
3 y	1.41	(1.12,1.78)	<0.01	1.38	(1.06,1.80)	0.02	1.19	(0.88, 1.62)	0.27
7 y	1.26	(1.10,1.45)	<0.01	1.21	(1.04,1.41)	0.01	1.10	(0.92, 1.31)	0.28
13 y	1.00	(0.90,1.11)	0.98	0.96	(0.82,1.13)	0.63	0.87	(0.73, 1.04)	0.12
Sugar intake									
3 y	1.57	(1.13, 2.18)	0.01	1.44	(1.02, 2.04)	0.04	1.37	(0.93, 2.01)	0.11
7 y	1.28	(1.05, 1.57)	0.02	1.23	(0.99, 1.53)	0.06	1.20	(0.95, 1.52)	0.12
13 y	0.96	(0.81, 1.14)	0.63	0.96	(0.79, 1.17)	0.67	0.97	(0.79, 1.19)	0.77
Starch intake									
3 y	1.33	(0.96, 1.85)	0.09	1.24	(0.86, 1.8)	0.25	0.92	(0.6, 1.41)	0.70
7 y	1.23	(1.00, 1.53)	0.05	1.14	(0.90, 1.43)	0.28	0.89	(0.68, 1.17)	0.40
13 y	1.09	(0.9, 1.32)	0.37	1.06	(0.79, 1.43)	0.70	0.73	(0.51, 1.04)	0.08
Protein intake									
3 y	2.78	(1.33,5.78)	0.01	2.60	(1.18,5.73)	0.02	1.67	(0.69, 4.00)	0.25
7 y	1.87	(1.23,2.84)	<0.01	1.75	(1.12,2.75)	0.02	1.30	(0.78, 2.15)	0.31
13 y	1.33	(0.95,1.87)	0.10	1.68	(1.02,2.77)	0.04	1.13	(0.65, 1.98)	0.67
Fat intake									
3 y	1.07	(1, 1.16)	0.06	1.05	(0.96, 1.15)	0.25	0.96	(0.87, 1.06)	0.43
7 y	1.04	(1, 1.08)	0.05	1.02	(0.98, 1.07)	0.34	0.97	(0.92, 1.02)	0.26
13 y	1.01	(0.98, 1.05)	0.44	1.01	(0.95, 1.06)	0.82	0.95	(0.89, 1.01)	0.11
MUFA intake									
3 y	1.26	(1.03, 1.54)	0.03	1.17	(0.94, 1.47)	0.17	0.94	(0.72, 1.22)	0.65
7 y	1.16	(1.03, 1.32)	0.02	1.10	(0.95, 1.27)	0.19	0.94	(0.80, 1.11)	0.45
13 y	1.08	(0.98, 1.18)	0.12	1.07	(0.94, 1.21)	0.33	0.94	(0.81, 1.09)	0.45
PUFA intake									
3 y	1.42	(1.07, 1.88)	0.02	1.30	(0.96, 1.77)	0.09	1.10	(0.77, 1.57)	0.59
7 y	1.21	(1.04, 1.41)	0.02	1.16	(0.98, 1.37)	0.08	1.06	(0.88, 1.29)	0.53
13 y	1.14	(0.94, 1.38)	0.20	1.09	(0.86, 1.37)	0.48	0.91	(0.69, 1.2)	0.52
SFA intake									
3 y	1.04	(0.92, 1.18)	0.53	1.01	(0.88, 1.15)	0.94	0.90	(0.77, 1.05)	0.17
7 y	1.03	(0.96, 1.11)	0.39	1.01	(0.93, 1.09)	0.87	0.94	(0.85, 1.03)	0.18
13 y	1.01	(0.93, 1.09)	0.85	0.98	(0.87, 1.1)	0.72	0.89	(0.78, 1.02)	0.09

Online Supporting Material

	USS Liver Stiffness								
	% Change[1]	95% CI	p	% Change[1]	95% CI	p	% Change[1]	95% CI	p
Energy intake									
3 y	1	(0,3)	0.04	1	(0, 3)	0.15	0	(-1, 2)	0.64
7 y	1	(0,2)	<0.01	1	(0, 1)	0.06	0	(0, 1)	0.35
13 y	0	(0,1)	0.39	0	(-1, 1)	0.43	0	(-1, 1)	0.78
Carbohydrate intake									
3 y	1	(0,1)	0.07	1	(0,1)	0.19	0	(-1, 1)	0.50
7 y	1	(0,1)	0.01	1	(0,1)	0.04	0	(0, 1)	0.16
13 y	0	(0,1)	0.33	0	(0,1)	0.26	0	(0, 1)	0.59
Sugar intake									
3 y	1	(0, 2)	0.07	1	(0, 2)	0.20	1	(-1, 2)	0.33
7 y	1	(0, 1)	0.08	0	(0, 1)	0.18	0	(0, 1)	0.28
13 y	0	(0, 1)	0.86	0	(0, 1)	0.70	0	(0, 1)	0.74
Starch intake									
3 y	1	(0, 2)	0.27	0	(-1, 2)	0.50	0	(-1, 1)	0.99
7 y	1	(0, 1)	0.03	1	(0, 1)	0.13	0	(0, 1)	0.47
13 y	1	(0, 1)	0.07	1	(0, 2)	0.06	1	(0, 2)	0.31
Protein intake									
3 y	0	(-2,3)	0.76	0	(-3,2)	0.75	-1	(-4, 1)	0.30
7 y	1	(-0,2)	0.33	0	(-0,2)	0.80	0	(-2, 1)	0.58
13 y	0	(-0,1)	0.99	0	(-2,1)	0.57	-1	(-3, 0)	0.12
Fat intake									
3 y	0	(0, 0)	0.13	0	(0, 0)	0.44	0	(0, 0)	0.87
7 y	0	(0, 0)	0.04	0	(0, 0)	0.25	0	(0, 0)	0.85
13 y	0	(0, 0)	0.50	0	(0, 0)	0.99	0	(0, 0)	0.40
MUFA intake									
3 y	0	(0, 1)	0.19	0	(-1, 1)	0.83	0	(-1, 0)	0.48
7 y	0	(0, 1)	0.05	0	(0, 1)	0.43	0	(-1, 0)	0.86
13 y	0	(0, 1)	0.30	0	(0, 0)	0.82	0	(-1, 0)	0.52
PUFA intake									
3 y	1	(0, 2)	0.07	1	(0, 2)	0.24	0	(-1, 1)	0.57
7 y	0	(0, 1)	0.16	0	(0, 1)	0.46	0	(-1, 1)	0.90
13 y	0	(0, 1)	0.18	0	(0, 1)	0.47	0	(-1, 1)	0.99
SFA intake									
3 y	0	(0, 1)	0.28	0	(0, 1)	0.59	0	(0, 0)	0.89
7 y	0	(0, 1)	0.17	0	(0, 1)	0.44	0	(0, 0)	0.98
13 y	0	(0, 1)	0.70	0	(0, 1)	0.95	0	(0, 0)	0.57

[1]Coefficients for energy intake are per 100 kcal increase in energy intake and coefficients absolute macronutrient intakes are per 10 gram increase in absolute macronutrient intake at ages 3, 7 and 13 y, except for coefficients corresponding to monounsaturated, polyunsaturated, saturated and total fat intakes which are per 1 gram increase.

[2] adjusted for sex, age at outcome assessment, maternal pre-pregnancy BMI, maternal age, social class, maternal education and parity.

[3] additionally adjusted for total body fatness at the time of outcome assessment.

MUFA – monounsaturated fatty acid; PUFA – polyunsaturated fatty acid; SFA – saturated fatty acid; USS – ultrasound scan

Online Supporting Material

Supplemental Table 4: Associations of energy intake and absolute macronutrient intakes at ages 3, 7 and 13 y with blood-based liver outcomes at mean age 17.8 y in the imputed dataset of eligible participants with blood-based liver outcome data (N=3059 at all ages)

	Unadjusted			Adjusted for potential confounders[2]			Adjusted for potential confounders and mediators[3]		
	ALT								
	% Change[1]	95% CI	p	% Change[1]	95% CI	p	% Change[1]	95% CI	p
Energy intake									
3 y	12	(10,15)	<0.01	7	(4,10)	<0.01	3	(0, 6)	0.03
7 y	6	(4,7)	<0.01	3	(2,5)	<0.01	2	(0, 3)	0.02
13 y	5	(4,6)	<0.01	4	(2,5)	<0.01	2	(0, 4)	0.02
Carbohydrate intake									
3 y	6	(5,8)	<0.01	3	(2,5)	<0.01	1	(0, 3)	0.10
7 y	3	(3,4)	<0.01	2	(0,3)	<0.01	1	(0, 2)	0.05
13 y	3	(3,4)	<0.01	2	(0,3)	<0.01	1	(0, 2)	0.02
Sugar intake									
3 y	5	(3, 7)	<0.01	2	(0, 4)	0.06	0	(-2, 2)	0.81
7 y	3	(0, 4)	<0.01	1	(0, 2)	0.05	0	(-1, 1)	0.72
13 y	3	(2, 4)	<0.01	0	(-1, 1)	0.36	0	(-1, 1)	0.99
Starch intake									
3 y	8	(6, 10)	<0.01	4	(2, 6)	<0.01	2	(0, 4)	0.03
7 y	5	(4, 6)	<0.01	3	(2, 4)	<0.01	2	(1, 3)	0.00
13 y	6	(5, 8)	<0.01	5	(3, 6)	<0.01	3	(1, 5)	0.00
Protein intake									
3 y	15	(10,20)	<0.01	9	(4,13)	<0.01	5	(0, 9)	0.03
7 y	9	(7,12)	<0.01	6	(3,8)	<0.01	3	(1, 6)	0.01
13 y	11	(9,13)	<0.01	6	(4,9)	<0.01	4	(1, 6)	0.01
Fat intake									
3 y	2	(1, 2)	0.00	1	(0, 1)	0.00	0	(0, 1)	0.16
7 y	1	(1, 1)	0.00	0	(0, 1)	0.00	0	(0, 0)	0.20
13 y	1	(1, 1)	0.00	0	(0, 1)	0.00	0	(0, 0)	0.41
MUFA intake									
3 y	5	(4, 6)	<0.01	2	(0, 4)	<0.01	1	(0, 2)	0.18
7 y	3	(2, 4)	<0.01	2	(0, 2)	<0.01	1	(0, 1)	0.13
13 y	3	(2, 3)	<0.01	1	(0, 2)	<0.01	0	(0, 1)	0.23
PUFA intake									
3 y	4	(3, 6)	<0.01	2	(0, 4)	0.03	1	(-1, 2)	0.55
7 y	2	(0, 3)	<0.01	1	(0, 2)	0.03	0	(-1, 1)	0.52
13 y	4	(3, 5)	<0.01	1	(0, 2)	0.03	0	(-1, 1)	0.59
SFA intake									
3 y	2	(2, 3)	<0.01	1	(0, 2)	<0.01	0	(0, 1)	0.23
7 y	1	(0, 2)	<0.01	1	(0, 1)	<0.01	0	(0, 1)	0.35
13 y	2	(2, 2)	<0.01	1	(0, 1)	0.02	0	(0, 1)	0.43

Online Supporting Material

	AST								
	% Change[1]	95% CI	p	% Change[1]	95% CI	p	% Change[1]	95% CI	p
Energy intake									
3 y	7	(6,9)	<0.01	1	(-0,3)	0.23	1	(-1, 2)	0.55
7 y	3	(2,3)	<0.01	1	(0,1)	0.09	1	(0, 1)	0.25
13 y	4	(3,4)	<0.01	1	(0,2)	0.04	1	(0, 2)	0.09
Carbohydrate intake									
3 y	4	(3,5)	<0.01	1	(0,2)	0.23	0	(-1, 2)	0.41
7 y	2	(0,2)	<0.01	1	(0,1)	0.02	1	(0, 1)	0.06
13 y	2	(2,3)	<0.01	1	(0,1)	<0.01	1	(0, 1)	0.00
Sugar intake									
3 y	3	(0, 4)	<0.01	0	(-1, 1)	0.74	0	(-1, 1)	0.97
7 y	2	(0, 2)	<0.01	1	(0, 1)	0.15	0	(0, 1)	0.23
13 y	3	(2, 3)	<0.01	1	(0, 1)	0.02	1	(0, 1)	0.03
Starch intake									
3 y	5	(3, 6)	<0.01	1	(-1, 2)	0.33	0	(-1, 2)	0.51
7 y	2	(2, 3)	<0.01	1	(0, 1)	0.11	1	(0, 1)	0.23
13 y	4	(4, 5)	<0.01	1	(0, 2)	0.03	1	(0, 2)	0.07
Protein intake									
3 y	6	(3,9)	<0.01	1	(-2,3)	0.60	0	(-3, 3)	0.99
7 y	4	(2,5)	<0.01	1	(-0,2)	0.36	0	(-1, 2)	0.67
13 y	7	(6,8)	<0.01	1	(-0,3)	0.30	1	(-1, 2)	0.57
Fat intake									
3 y	1	(1, 1)	0.00	0	(0, 0)	0.90	0	(0, 0)	0.62
7 y	0	(0, 0)	0.00	0	(0, 0)	0.75	0	(0, 0)	0.79
13 y	1	(1, 1)	0.00	0	(0, 0)	0.87	0	(0, 0)	0.55
MUFA intake									
3 y	2	(0, 3)	<0.01	0	(-1, 1)	0.63	0	(-1, 0)	0.27
7 y	1	(0, 2)	<0.01	0	(0, 1)	0.99	0	(-1, 0)	0.57
13 y	2	(0, 2)	<0.01	0	(-1, 1)	0.69	0	(-1, 0)	0.40
PUFA intake									
3 y	2	(0, 3)	<0.01	0	(-1, 1)	0.92	0	(-1, 1)	0.81
7 y	1	(0, 1)	0.01	0	(-1, 1)	0.99	0	(-1, 0)	0.65
13 y	2	(0, 3)	<0.01	0	(-1, 1)	0.33	-1	(-1, 0)	0.18
SFA intake									
3 y	1	(0, 1)	<0.01	0	(0, 1)	0.75	0	(0, 0)	0.89
7 y	1	(0, 1)	<0.01	0	(0, 1)	0.56	0	(0, 0)	0.86
13 y	1	(0, 2)	<0.01	0	(0, 1)	0.68	0	(0, 0)	0.86

Online Supporting Material

	GGT								
	% Change[1]	95% CI	p	% Change[1]	95% CI	p	% Change[1]	95% CI	p
Energy intake									
3 y	12	(10,14)	<0.01	5	(2,7)	<0.01	1	(-2, 3)	0.56
7 y	5	(4,6)	<0.01	2	(0,3)	<0.01	0	(-1, 1)	0.69
13 y	6	(5,7)	<0.01	3	(0,4)	<0.01	1	(-1, 2)	0.47
Carbohydrate intake									
3 y	7	(6,8)	<0.01	3	(2,4)	<0.01	1	(0, 3)	0.03
7 y	4	(3,4)	<0.01	2	(0,2)	<0.01	1	(0, 2)	0.04
13 y	4	(3,4)	<0.01	2	(0,2)	<0.01	1	(0, 2)	0.01
Sugar intake									
3 y	7	(6, 9)	<0.01	4	(2, 5)	<0.01	2	(1, 4)	0.00
7 y	4	(3, 5)	<0.01	2	(0, 3)	<0.01	1	(1, 2)	0.00
13 y	4	(3, 5)	<0.01	2	(0, 2)	<0.01	1	(0, 2)	0.01
Starch intake									
3 y	6	(5, 8)	<0.01	1	(-1, 3)	0.24	-1	(-2, 1)	0.40
7 y	3	(2, 5)	<0.01	1	(0, 2)	0.09	0	(-1, 1)	0.43
13 y	6	(5, 7)	<0.01	2	(0, 3)	0.02	0	(-1, 1)	0.90
Protein intake									
3 y	12	(9,16)	<0.01	5	(0,8)	0.01	1	(-3, 4)	0.65
7 y	7	(5,9)	<0.01	3	(0,5)	<0.01	0	(-2, 2)	0.70
13 y	11	(10,13)	<0.01	4	(2,6)	<0.01	1	(-1, 3)	0.30
Fat intake									
3 y	2	(1, 2)	0.00	0	(0, 1)	0.14	0	(-1, 0)	0.09
7 y	1	(0, 1)	0.00	0	(0, 0)	0.30	0	(0, 0)	0.03
13 y	1	(1, 1)	0.00	0	(0, 0)	0.81	0	(-1, 0)	0.01
MUFA intake									
3 y	4	(3, 5)	<0.01	1	(0, 2)	0.16	-1	(-2, 0)	0.12
7 y	2	(0, 2)	<0.01	0	(0, 1)	0.53	-1	(-1, 0)	0.01
13 y	2	(2, 3)	<0.01	0	(-1, 1)	0.96	-1	(-1, 0)	0.01
PUFA intake									
3 y	3	(2, 5)	<0.01	0	(-1, 1)	0.93	-1	(-3, 0)	0.08
7 y	1	(0, 2)	<0.01	0	(-1, 1)	0.96	-1	(-1, 0)	0.07
13 y	3	(2, 4)	<0.01	0	(-1, 1)	0.84	-1	(-2, 0)	0.03
SFA intake									
3 y	2	(0, 2)	<0.01	1	(0, 1)	0.06	0	(-1, 0)	0.66
7 y	1	(0, 1)	<0.01	0	(0, 1)	0.15	0	(0, 0)	0.40
13 y	2	(2, 2)	<0.01	0	(0, 1)	0.44	0	(-1, 0)	0.32

[1]Coefficients for energy intake are per 100 kcal increase in energy intake and coefficients absolute macronutrient intakes are per 10 gram increase in absolute macronutrient intake at ages 3, 7 and 13 y, except for coefficients corresponding to monounsaturated, polyunsaturated, saturated and total fat intakes which are per 1 gram increase.

[2] adjusted for sex, age at outcome assessment, maternal pre-pregnancy BMI, maternal age, social class, maternal education and parity.

[3] additionally adjusted for total body fatness at the time of outcome assessment.

ALT – alanine aminotransferase; AST – aspartate aminotransferase; GGT- gamma glutamyl transferase; MUFA – monounsaturated fatty acid; PUFA – polyunsaturated fatty acid; SFA – saturated fatty acid.

Online Supporting Material

Supplemental Table 5: Associations of energy intake and energy-adjusted macronutrient intakes at ages 3, 7 and 13 y with USS liver outcomes at mean age 17.8 y in the imputed dataset of eligible participants with USS data (N=1786 at all ages)

	Unadjusted			Adjusted for potential confounders[2]			Adjusted for potential confounders and mediators[3]		
	USS-measured liver fat								
	OR[1]	95% CI	p	OR[1]	95% CI	p	OR[1]	95% CI	p
Energy intake									
3 y	1.77	(1.23,2.57)	0.00	1.79	(1.14,2.79)	0.01	1.17	(0.68,1.99)	0.57
7 y	1.39	(1.15,1.67)	0.00	1.30	(1.06,1.60)	0.01	0.60	(0.17,2.10)	0.43
13 y	1.07	(0.91,1.26)	0.38	1.12	(0.84,1.49)	0.45	0.46	(0.24,0.89)	0.02
Carbohydrate intake									
3 y	1.13	(0.70,1.84)	0.61	1.12	(0.68, 1.84)	0.66	1.35	(0.77, 2.38)	0.30
7 y	1.10	(0.81,1.50)	0.54	1.10	(0.8, 1.52)	0.54	1.25	(0.88, 1.77)	0.22
13 y	0.86	(0.66,1.13)	0.29	0.88	(0.66, 1.17)	0.39	0.97	(0.7, 1.35)	0.86
Sugar intake									
3 y	1.29	(0.82, 2.03)	0.27	1.26	(0.80, 1.98)	0.32	1.50	(0.92, 2.45)	0.10
7 y	1.09	(0.83, 1.43)	0.54	1.12	(0.85, 1.47)	0.43	1.32	(0.98, 1.78)	0.07
13 y	0.90	(0.72, 1.13)	0.38	0.96	(0.77, 1.22)	0.76	1.13	(0.89, 1.43)	0.30
Starch intake									
3 y	0.87	(0.58, 1.31)	0.51	0.90	(0.59, 1.38)	0.64	0.83	(0.52, 1.32)	0.43
7 y	0.95	(0.69, 1.31)	0.75	0.93	(0.66, 1.3)	0.66	0.82	(0.57, 1.19)	0.30
13 y	1.00	(0.73, 1.38)	1.00	0.95	(0.68, 1.33)	0.76	0.77	(0.53, 1.14)	0.19
Protein intake									
3 y	1.40	(0.58,3.34)	0.45	1.48	(0.59,3.73)	0.41	1.61	(0.57, 4.58)	0.37
7 y	1.28	(0.70,2.31)	0.42	1.39	(0.74,2.61)	0.31	1.38	(0.69, 2.79)	0.36
13 y	1.51	(0.82,2.80)	0.19	1.79	(0.93,3.46)	0.08	1.66	(0.79, 3.52)	0.18
Fat intake									
3 y	0.57	(0.16,1.98)	0.37	0.55	(0.15,1.99)	0.36	0.32	(0.08, 1.28)	0.11
7 y	0.66	(0.31,1.43)	0.29	0.60	(0.27,1.35)	0.22	0.45	(0.19, 1.06)	0.07
13 y	1.09	(0.54,2.23)	0.81	0.93	(0.43,1.99)	0.85	0.77	(0.32, 1.84)	0.55
MUFA intake									
3 y	1.03	(0.73, 1.45)	0.88	0.93	(0.65, 1.34)	0.70	0.80	(0.55, 1.17)	0.25
7 y	0.99	(0.78, 1.24)	0.92	0.93	(0.73, 1.18)	0.53	0.83	(0.65, 1.07)	0.16
13 y	1.16	(0.97, 1.38)	0.10	1.12	(0.92, 1.36)	0.25	1.07	(0.86, 1.34)	0.53
PUFA intake									
3 y	1.15	(0.86, 1.54)	0.35	1.15	(0.85, 1.56)	0.35	1.21	(0.85, 1.71)	0.28
7 y	1.09	(0.88, 1.33)	0.44	1.07	(0.87, 1.33)	0.51	1.08	(0.84, 1.37)	0.56
13 y	1.11	(0.91, 1.36)	0.29	1.07	(0.86, 1.32)	0.54	1.00	(0.78, 1.27)	0.98
SFA intake									
3 y	0.90	(0.78, 1.04)	0.15	0.91	(0.78, 1.05)	0.18	0.86	(0.73, 1.01)	0.06
7 y	0.93	(0.84, 1.03)	0.18	0.93	(0.84, 1.04)	0.20	0.91	(0.81, 1.02)	0.12
13 y	0.96	(0.84, 1.09)	0.52	0.95	(0.82, 1.09)	0.43	0.93	(0.8, 1.1)	0.41

Online Supporting Material

	USS Liver Stiffness								
	% Change[1]	95% CI	p	% Change[1]	95% CI	p	% Change[1]	95% CI	p
Energy intake									
3 y	1	(0,3)	0.04	1	(0,3)	0.15	0	(-1,2)	0.64
7 y	1	(0,2)	0.00	1	(0,1)	0.06	3	(0,7)	0.04
13 y	0	(0,1)	0.39	0	(-1,1)	0.43	-3	(-5,-1)	0.01
Carbohydrate intake									
3 y	0	(-1,2)	0.63	1	(-1,2)	0.52	1	(-1, 2)	0.38
7 y	0	(-1,1)	0.61	0	(-1,1)	0.51	0	(-1, 1)	0.37
13 y	1	(0,1)	0.24	1	(0,2)	0.13	1	(0, 2)	0.07
Sugar intake									
3 y	1	(-1, 2)	0.22	1	(-1, 2)	0.28	1	(0, 2)	0.20
7 y	0	(-1, 1)	0.60	0	(-1, 1)	0.51	0	(0, 1)	0.32
13 y	0	(-1, 1)	0.80	0	(-1, 1)	0.74	0	(0, 1)	0.38
Starch intake									
3 y	0	(-2, 1)	0.54	0	(-1, 1)	0.75	0	(-1, 1)	0.72
7 y	0	(-1, 1)	0.77	0	(-1, 1)	0.75	0	(-1, 1)	0.87
13 y	1	(0, 2)	0.05	1	(0, 2)	0.09	1	(0, 2)	0.18
Protein intake									
3 y	-2	(-5,1)	0.14	-2	(-5, 1)	0.11	-2	(-5, 1)	0.11
7 y	-1	(-3,0)	0.15	-1	(-3, 0)	0.14	-1	(-3, 0)	0.12
13 y	-2	(-3,0)	0.09	-1	(-3, 0)	0.13	-2	(-4, 0)	0.07
Fat intake									
3 y	1	(-3, 5)	0.69	0	(-3, 4)	0.85	0	(-4, 4)	0.92
7 y	0	(-2, 3)	0.85	0	(-2, 2)	0.97	0	(-3, 2)	0.80
13 y	-1	(-3, 2)	0.56	-1	(-3, 1)	0.29	-1	(-3, 1)	0.24
MUFA intake									
3 y	0	(-1, 1)	0.57	0	(-1, 1)	0.81	0	(-1, 1)	0.56
7 y	0	(-1, 1)	0.72	0	(-1, 1)	0.70	0	(-1, 0)	0.50
13 y	0	(0, 1)	0.75	0	(-1, 0)	0.72	0	(-1, 0)	0.53
PUFA intake									
3 y	0	(-1, 1)	0.97	0	(-1, 1)	0.93	0	(-1, 1)	0.99
7 y	0	(-1, 1)	0.82	0	(-1, 1)	0.93	0	(-1, 1)	0.98
13 y	0	(0, 1)	0.43	0	(-1, 1)	0.87	0	(-1, 1)	0.99
SFA intake									
3 y	0	(0, 0)	0.78	0	(0, 0)	0.80	0	(0, 0)	0.96
7 y	0	(0, 0)	0.75	0	(0, 0)	0.79	0	(0, 0)	0.75
13 y	0	(-1, 0)	0.54	0	(-1, 0)	0.55	0	(0, 0)	0.61

[1]Coefficients for energy intake are per 100 kcal increase in energy intake and coefficients absolute macronutrient intakes are per 10 gram increase in absolute macronutrient intake at ages 3, 7 and 13 y, except for coefficients corresponding to monounsaturated, polyunsaturated, saturated and total fat intakes which are per 1 gram increase.
[2] adjusted for sex, age at outcome assessment, maternal pre-pregnancy BMI, maternal age, social class, maternal education and parity.
[3] additionally adjusted for total body fatness at the time of outcome assessment.
MUFA – monounsaturated fatty acid; PUFA – polyunsaturated fatty acid; SFA – saturated fatty acid; USS – ultrasound scan

Online Supporting Material

Supplemental Table 6: Associations of energy intake and energy-adjusted macronutrient intakes at ages 3, 7 and 13 y with blood-based liver outcomes at mean age 17.8 y in the imputed dataset of eligible participants with blood-based liver outcome data (N=3059 at all ages)

	Unadjusted			Adjusted for potential confounders[2]			Adjusted for potential confounders and mediators[3]		
	ALT								
	% Change[1]	95% CI	p	% Change[1]	95% CI	p	% Change[1]	95% CI	p
Energy intake									
3 y	12	(10,15)	0.00	7	(4,10)	0.00	3	(0,6)	0.03
7 y	6	(4,7)	0.00	3	(2,5)	0.00	2	(-3,8)	0.42
13 y	5	(4,6)	0.00	4	(2,5)	0.00	1	(-2,4)	0.43
Carbohydrate intake									
3 y	0	(-3, 2)	0.86	-1	(-3, 2)	0.62	0	(-3, 2)	0.99
7 y	0	(-2, 2)	0.95	0	(-2, 2)	0.89	0	(-1, 2)	0.87
13 y	0	(-1, 2)	0.60	1	(-1, 2)	0.43	1	(-1, 2)	0.34
Sugar intake									
3 y	-1	(-4, 1)	0.22	-1	(-4, 1)	0.26	-1	(-3, 1)	0.31
7 y	-1	(-3, 0)	0.09	-1	(-2, 0)	0.16	-1	(-2, 1)	0.26
13 y	-1	(-2, 0)	0.02	-1	(-2, 0)	0.06	-1	(-2, 0)	0.14
Starch intake									
3 y	1	(-1, 3)	0.24	1	(-1, 3)	0.37	1	(-1, 3)	0.26
7 y	2	(0, 4)	0.03	1	(0, 3)	0.09	1	(0, 3)	0.10
13 y	3	(2, 5)	0.00	3	(1, 5)	0.00	3	(1, 4)	0.00
Protein intake									
3 y	0	(-4, 5)	0.94	2	(-3, 6)	0.53	2	(-3, 6)	0.45
7 y	2	(-1, 5)	0.19	2	(-1, 6)	0.14	2	(-1, 5)	0.19
13 y	4	(1, 7)	0.02	3	(0, 7)	0.04	3	(-1, 6)	0.11
Fat intake									
3 y	1	(-3, 5)	0.69	0	(-3, 4)	0.85	0	(-4, 4)	0.98
7 y	0	(-2, 3)	0.85	0	(-2, 2)	0.97	0	(-3, 2)	0.80
13 y	-1	(-3, 2)	0.56	-1	(-3, 1)	0.29	-1	(-3, 1)	0.24
MUFA intake									
3 y	1	(-1, 2)	0.43	0	(-1, 2)	0.61	0	(-2, 2)	0.92
7 y	0	(-1, 1)	0.68	0	(-1, 1)	0.80	0	(-1, 1)	0.88
13 y	0	(-1, 1)	0.84	0	(-1, 1)	0.40	-1	(-1, 0)	0.22
PUFA intake									
3 y	-1	(-2, 1)	0.36	0	(-2, 1)	0.62	0	(-2, 1)	0.65
7 y	0	(-1, 1)	0.43	0	(-1, 1)	0.63	0	(-1, 1)	0.58
13 y	0	(-1, 1)	0.87	0	(-1, 1)	0.83	0	(-1, 1)	0.57
SFA intake									
3 y	0	(0, 1)	0.36	0	(0, 1)	0.49	0	(-1, 1)	0.83
7 y	0	(-1, 0)	0.66	0	(-1, 0)	0.68	0	(-1, 0)	0.63
13 y	0	(-1, 0)	0.24	0	(-1, 0)	0.23	0	(-1, 0)	0.45

Online Supporting Material

	AST								
	% Change[1]	95% CI	p	% Change[1]	95% CI	p	% Change[1]	95% CI	p
Energy intake									
3 y	7	(6,9)	0.00	1	(-1,3)	0.23	1	(-1,2)	0.55
7 y	3	(2,3)	0.00	1	(0,1)	0.09	4	(1,8)	0.02
13 y	4	(3,4)	0.00	1	(0,2)	0.04	1	(-1,3)	0.53
Carbohydrate intake									
3 y	1	(-1,3)	0.18	1	(-1,2)	0.49	1	(-1, 2)	0.38
7 y	1	(0,2)	0.05	1	(0,2)	0.13	1	(0, 2)	0.10
13 y	1	(0,2)	0.00	1	(0,2)	0.01	1	(0, 2)	0.00
Sugar intake									
3 y	-1	(-2, 1)	0.51	0	(-2, 1)	0.89	0	(-2, 1)	0.94
7 y	0	(-1, 1)	0.89	0	(-1, 1)	0.53	0	(-1, 1)	0.47
13 y	0	(0, 1)	0.38	1	(0, 1)	0.17	1	(0, 1)	0.14
Starch intake									
3 y	1	(0, 3)	0.08	1	(-1, 2)	0.43	1	(-1, 2)	0.38
7 y	1	(0, 2)	0.07	0	(-1, 2)	0.40	0	(-1, 2)	0.40
13 y	1	(0, 2)	0.02	1	(0, 2)	0.19	1	(0, 2)	0.23
Protein intake									
3 y	-2	(-5,1)	0.28	0	(-3,3)	0.92	0	(-3, 3)	0.99
7 y	-1	(-3,2)	0.56	0	(-2,2)	0.78	0	(-2, 2)	0.70
13 y	1	(-1,3)	0.48	0	(-2,2)	0.75	-1	(-3, 2)	0.61
Fat intake									
3 y	-1	(-5, 3)	0.60	-1	(-5, 3)	0.54	-2	(-6, 3)	0.42
7 y	-3	(-5, 0)	0.06	-2	(-5, 1)	0.14	-2	(-5, 1)	0.12
13 y	-5	(-7, -2)	0.00	-4	(-6, -1)	0.00	-6	(-1, -3)	0.00
MUFA intake									
3 y	-1	(-2, 0)	0.15	-1	(-2, 1)	0.26	-1	(-2, 0)	0.18
7 y	-1	(-2, 0)	0.07	-1	(-1, 0)	0.14	-1	(-1, 0)	0.11
13 y	-1	(-1, 0)	0.00	-1	(-1, 0)	0.00	-1	(-1, 0)	0.00
PUFA intake									
3 y	0	(-1, 1)	0.60	0	(-1, 1)	0.90	0	(-1, 1)	0.89
7 y	0	(-1, 0)	0.29	0	(-1, 0)	0.46	0	(-1, 0)	0.44
13 y	-1	(-2, 0)	0.00	-1	(-2, 0)	0.01	-1	(-2, 0)	0.01
SFA intake									
3 y	0	(0, 1)	0.73	0	(0, 0)	0.98	0	(0, 0)	0.90
7 y	0	(0, 0)	0.56	0	(0, 0)	0.60	0	(0, 0)	0.59
13 y	0	(-1, 0)	0.18	0	(-1, 0)	0.26	0	(-1, 0)	0.32

Online Supporting Material

	GGT								
	% Change[1]	95% CI	p	% Change[1]	95% CI	p	% Change[1]	95% CI	p
Energy intake									
3 y	12	(10,14)	0.00	5	(2,7)	0.00	1	(-2,3)	0.56
7 y	5	(4,6)	0.00	2	(1,3)	0.00	-1	(-6,3)	0.50
13 y	6	(5,7)	0.00	3	(1,4)	0.00	1	(-1,4)	0.29
Carbohydrate intake									
3 y	3	(1,5)	0.01	2	(0,5)	0.03	3	(1, 5)	0.01
7 y	2	(1,4)	0.00	2	(0,3)	0.01	2	(1, 3)	0.00
13 y	2	(1,3)	0.00	2	(1,3)	0.00	2	(1, 3)	0.00
Sugar intake									
3 y	3	(1, 5)	0.01	3	(1, 5)	0.00	3	(1, 5)	0.00
7 y	2	(0, 3)	0.01	2	(1, 3)	0.00	2	(1, 3)	0.00
13 y	1	(0, 2)	0.08	1	(0, 2)	0.02	1	(0, 2)	0.00
Starch intake									
3 y	-1	(-2, 1)	0.53	-1	(-3, 1)	0.17	-1	(-3, 1)	0.22
7 y	0	(-1, 1)	0.99	-1	(-2, 1)	0.36	-1	(-2, 1)	0.29
13 y	1	(0, 2)	0.16	0	(-1, 2)	0.55	0	(-1, 1)	0.93
Protein intake									
3 y	-2	(-6,2)	0.27	0	(-4,4)	0.93	0	(-4, 4)	0.96
7 y	0	(-3,3)	0.95	1	(-2,3)	0.67	0	(-2, 3)	0.82
13 y	3	(0,6)	0.03	2	(-1,5)	0.20	1	(-2, 4)	0.46
Fat intake									
3 y	-5	(-10, 0)	0.05	-6	(-11, -1)	0.03	-8	(-12, -3)	0.00
7 y	-6	(-1, -2)	0.00	-5	(-8, -2)	0.00	-6	(-1, -2)	0.00
13 y	-7	(-10, -4)	0.00	-6	(-1, -4)	0.00	-6	(-1, -3)	0.00
MUFA intake									
3 y	-2	(-3, 0)	0.04	-2	(-3, 0)	0.04	-2	(-4, -1)	0.00
7 y	-2	(-2, -1)	0.00	-1	(-2, 0)	0.00	-2	(-3, -1)	0.00
13 y	-1	(-2, -1)	0.00	-1	(-2, -1)	0.00	-2	(-2, -1)	0.00
PUFA intake									
3 y	-1	(-3, 0)	0.03	-1	(-2, 0)	0.08	-1	(-2, 0)	0.08
7 y	-1	(-2, 0)	0.03	-1	(-2, 0)	0.06	-1	(-2, 0)	0.04
13 y	-1	(-2, 0)	0.01	-1	(-2, 0)	0.06	-1	(-2, 0)	0.02
SFA intake									
3 y	0	(-1, 1)	0.85	0	(-1, 1)	0.86	0	(-1, 0)	0.47
7 y	0	(-1, 0)	0.37	0	(-1, 0)	0.37	0	(-1, 0)	0.32
13 y	-1	(-1, 0)	0.04	-1	(-1, 0)	0.04	0	(-1, 0)	0.12

[1]Coefficients for energy intake are per 100 kcal increase in energy intake and coefficients absolute macronutrient intakes are per 10 gram increase in absolute macronutrient intake at ages 3, 7 and 13 y, except for coefficients corresponding to monounsaturated, polyunsaturated, saturated and total fat intakes which are per 1 gram increase.
[2] adjusted for sex, age at outcome assessment, maternal pre-pregnancy BMI, maternal age, social class, maternal education and parity.
[3] additionally adjusted for total body fatness at the time of outcome assessment.
ALT – alanine aminotransferase; AST – aspartate aminotransferase; GGT- gamma glutamyl transferase; MUFA – monounsaturated fatty acid; PUFA – polyunsaturated fatty acid; SFA – saturated fatty acid.

Online Supporting Material

Supplemental Table 7: Associations of energy intake at ages 3, 7 and 13 y with continuous liver outcomes at mean age 17.8 y in participants classified as plausible reporters.

	Unadjusted			Adjusted for potential confounders[2]			Adjusted for potential confounders and mediators[3]		
	Energy intake								
	% Change[1]	95% CI	p	% Change[1]	95% CI	p	% Change[1]	95% CI	p
USS Liver stiffness[4]									
3 y	1	(-2,4)	0.48	-2	(-5,2)	0.33	-1	(0.95, 3)	0.68
7 y	0	(-1,2)	0.71	-1	(-2,1)	0.46	0	(-2, 2)	0.91
13 y	1	(0,2)	0.05	0	(-2,2)	0.71	0	(-2, 3)	0.77
ALT[5]									
3 y	13	(8,18)	<0.01	6	(0,12)	0.05	5	(-1, 12)	0.12
7 y	6	(4,9)	<0.01	4	(1,6)	0.01	3	(0, 7)	0.03
13 y	6	(4,8)	<0.01	6	(2,9)	<0.01	6	(2, 10)	0.01
AST[5]									
3 y	7	(4,11)	<0.01	-1	(-5,4)	0.77	1	(-3, 6)	0.80
7 y	3	(1,5)	<0.01	0	(-2,2)	0.82	1	(-1, 3)	0.39
13 y	4	(3,6)	<0.01	1	(-1,4)	0.31	3	(0, 5)	0.08
GGT[5]									
3 y	11	(6,16)	<0.01	3	(-3,9)	0.35	2	(-3, 9)	0.47
7 y	5	(3,8)	<0.01	2	(-1,5)	0.18	2	(-1, 5)	0.26
13 y	6	(4,8)	<0.01	5	(2,8)	<0.01	5	(1, 9)	0.01

[1]Coefficients for energy intake are per 100 kcal increase in energy intake

[2] adjusted for sex, age at outcome assessment, maternal pre-pregnancy BMI, maternal age, social class, maternal education and parity.

[3] additionally adjusted for total body fatness at the time of outcome assessment.

[4] N=267 for USS liver outcomes at all ages

[5] N=483 for blood-based liver outcomes at all ages

ALT – alanine aminotransferase; AST – aspartate aminotransferase; GGT- gamma glutamyl transferase; USS – ultrasound scan

Online Supporting Material

Supplemental Table 8: Complete case associations of energy intake at ages 3, 7 and 13 y with USS and blood-based liver outcomes at mean age 17.8 y (i.e. with no missing data)

	Unadjusted			Adjusted for potential confounders[2]			Adjusted for potential confounders and mediators[3]		
	Energy intake								
	OR[1]	95% CI	p	OR[1]	95% CI	p	OR[1]	95% CI	p
USS-measured liver fat[4]									
3 y	2.20	(1.35,3.57)	<0.01	2.49	(1.36,4.54)	<0.01	1.25	(0.61,2.59)	0.54
7 y	1.54	(1.20,1.98)	<0.01	1.53	(1.15,2.04)	<0.01	1.08	(0.76,1.54)	0.65
13 y	1.15	(0.93,1.41)	0.20	1.23	(0.84,1.82)	0.28	0.80	(0.53,1.22)	0.31
	% Change[1]	95% CI	p	% Change[1]	95% CI	p	% Change[1]	95% CI	p
USS liver stiffness[4]									
3 y	1	(0,3)	0.05	1	(-1,3)	0.19	1	(-1,3)	0.46
7 y	1	(0,2)	0.01	1	(0,2)	0.08	1	(0,1)	0.23
13 y	0	(0,1)	0.16	1	(0,2)	0.31	0	(-1,1)	0.70
ALT[5]									
3 y	13	(11,16)	<0.01	8	(5,11)	<0.01	5	(2, 8)	<0.01
7 y	6	(5,8)	<0.01	4	(3,5)	<0.01	2	(1,4)	<0.01
13 y	5	(5,6)	<0.01	5	(3,7)	<0.01	3	(1,5)	<0.01
AST[5]									
3 y	8	(6,10)	<0.01	3	(1,4)	0.01	3	(0,5)	0.02
7 y	3	(2,4)	<0.01	1	(1,2)	<0.01	1	(0,2)	<0.01
13 y	4	(3,5)	<0.01	2	(1,3)	<0.01	2	(1,3)	<0.01
GGT[5]									
3 y	13	(11,15)	<0.01	5	(3,8)	<0.01	2	(-1,4)	0.21
7 y	5	(4,6)	<0.01	2	(1,4)	<0.01	1	(-1,2)	0.34
13 y	6	(5,7)	<0.01	3	(1,4)	<0.01	1	(-1,2)	0.28

[1]Coefficients for energy intake are per 100 kcal increase in energy intake

[2] adjusted for sex, age at outcome assessment, maternal pre-pregnancy BMI, maternal age, social class, maternal education and parity.

[3] additionally adjusted for total body fatness at the time of outcome assessment

[4] N=1192 for USS liver outcomes at all ages

[5] N=2338 for blood-based liver outcomes at all ages

ALT – alanine aminotransferase; AST – aspartate aminotransferase; GGT- gamma glutamyl transferase; USS – ultrasound scan

Online Supporting Material

Supplemental Table 9: Associations of energy intake at ages 3, 7 and 13 y with USS and blood-based liver outcomes at mean age 17.8 y in participants with at least 2 measures of dietary intake

	Unadjusted			Adjusted for potential confounders[2]			Adjusted for potential confounders and mediators[3]		
	Energy intake								
	OR[1]	95% CI	p	OR[1]	95% CI	p	OR[1]	95% CI	p
USS-measured liver fat[4]									
3 y	1.84	(1.26,2.69)	<0.01	1.82	(1.15,2.87)	0.01	1.16	(0.67, 2.00)	0.60
7 y	1.40	(1.16,1.70)	<0.01	1.32	(1.06,1.63)	0.01	1.05	(0.81, 1.36)	0.72
13 y	1.10	(0.93,1.29)	0.28	1.12	(0.84,1.51)	0.44	0.81	(0.58, 1.13)	0.21
	% Change[1]	95% CI	p	% Change[1]	95% CI	p	% Change[1]	95% CI	p
USS liver stiffness[4]									
3 y	1	(0,2)	0.06	1	(-1,2)	0.22	0	(-1, 2)	0.69
7 y	1	(0,2)	0.01	1	(0,1)	0.08	0	(0, 1)	0.36
13 y	0	(0,1)	0.37	0	(-1,1)	0.52	0	(-1, 1)	0.78
ALT[5]									
3 y	12	(10,15)	<0.01	7	(4,10)	<0.01	3	(0, 6)	0.02
7 y	5	(4,7)	<0.01	3	(2,5)	<0.01	2	(0, 3)	0.01
13 y	5	(4,6)	<0.01	4	(2,5)	<0.01	2	(0, 4)	0.01
AST[5]									
3 y	7	(5,8)	<0.01	1	(-1,3)	0.26	1	(-1, 3)	0.46
7 y	3	(2,3)	<0.01	1	(0,1)	0.10	1	(0, 1)	0.19
13 y	4	(3,4)	<0.01	1	(0,2)	0.05	1	(0, 2)	0.07
GGT[5]									
3 y	12	(10,14)	<0.01	4	(2,7)	<0.01	1	(-2, 3)	0.53
7 y	5	(4,6)	<0.01	2	(1,3)	<0.01	0	(-1, 1)	0.63
13 y	6	(5,7)	<0.01	2	(1,4)	<0.01	1	(-1, 2)	0.39

[1]Coefficients for energy intake are per 100 kcal increase in energy intake

[2] adjusted for sex, age at outcome assessment, maternal pre-pregnancy BMI, maternal age, social class, maternal education and parity.

[3] additionally adjusted for total body fatness at the time of outcome assessment

[4] N=1734 for USS liver outcomes at all ages

[5] N=2994 for blood-based liver outcomes at all ages

ALT – alanine aminotransferase; AST – aspartate aminotransferase; GGT- gamma glutamyl transferase; USS – ultrasound scan

Online Supporting Material

Supplemental Table 10: Associations of energy intake at ages 3, 7 and 13 y with USS and blood-based liver outcomes at mean age 17.8 y in participants with at least 1 measure of dietary intake in each age period defined by the linear-spline model

	Unadjusted			Adjusted for potential confounders[2]			Adjusted for potential confounders and mediators[3]		
	Energy intake								
	OR[1]	95% CI	p	OR[1]	95% CI	p	OR[1]	95% CI	p
USS-measured liver fat[4]									
3 y	1.86	(1.19,2.90)	0.01	1.95	(1.13,3.36)	0.02	1.21	(0.64, 2.27)	0.56
7 y	1.42	(1.14,1.77)	<0.01	1.36	(5,1.75)	0.02	1.08	(0.8, 1.46)	0.63
13 y	1.11	(0.91,1.35)	0.31	1.17	(0.81,1.67)	0.40	0.86	(0.58, 1.27)	0.44
	% Change[1]	95% CI	p	% Change[1]	95% CI	p	% Change[1]	95% CI	p
USS liver stiffness[4]									
3 y	1	(0,2)	0.08	1	(-1,2)	0.30	0	(-1, 2)	0.61
7 y	1	(0,2)	0.01	1	(0,1)	0.10	0	(0, 1)	0.26
13 y	0	(0,1)	0.23	0	(-1,1)	0.38	0	(-1, 1)	0.76
ALT[5]									
3 y	13	(10,15)	<0.01	7	(4,10)	<0.01	4	(1, 7)	0.01
7 y	6	(5,7)	<0.01	4	(2,5)	<0.01	2	(1, 4)	<0.01
13 y	5	(4,6)	<0.01	4	(2,6)	<0.01	2	(1, 4)	0.01
AST[5]									
3 y	7	(5,9)	<0.01	1	(-1,3)	0.23	1	(-1, 3)	0.33
7 y	3	(2,4)	<0.01	1	(0,2)	0.08	1	(0, 2)	0.12
13 y	4	(3,4)	<0.01	1	(0,2)	0.08	1	(0, 2)	0.08
GGT[5]									
3 y	12	(10,14)	<0.01	5	(2,7)	<0.01	2	(-1, 4)	0.19
7 y	5	(4,6)	<0.01	2	(1,3)	<0.01	1	(0, 2)	0.24
13 y	6	(5,7)	<0.01	3	(1,4)	<0.01	1	(0, 3)	0.08

[1]Coefficients for energy intake are per 100 kcal increase in energy intake
[2] adjusted for sex, age at outcome assessment, maternal pre-pregnancy BMI, maternal age, social class, maternal education and parity.
[3] additionally adjusted for total body fatness at the time of outcome assessment
[4] N=1431 for USS liver outcomes at all ages
[5] N=2534 for blood-based liver outcomes at all ages
ALT – alanine aminotransferase; AST – aspartate aminotransferase; GGT- gamma glutamyl transferase; USS – ultrasound scan

Online Supporting Material

Supplemental Table 11: Comparing results from the complete case model with results from the bivariate multilevel model for associations of energy intake at ages 3 and change in energy intake from 3-7 and 7-13 y with USS liver stiffness (n=1992) and blood-based (n=2338) liver outcomes at mean age 17.8 y

	Unadjusted						Adjusted for potential confounders[2]					
	Bivariate model results			Complete case model results			Bivariate model results			Complete case model results		
	% Change[1]	95% CI	p	% Change[1]	95% CI	p	% Change[1]	95% CI	p	% Change[1]	95% CI	p
USS liver stiffness[3]												
3 y	-1	(-3,2)	0.65	2	(0,3)	0.05	-1	(-3,1)	0.43	1	(-1,3)	0.19
3-7 y	22	(5,40)	0.01	8	(2,14)	0.01	22	(3,44)	0.02	6	(0, 12)	0.04
7-13 y	-10	(-19,0)	0.04	-10	(-18,-2)	0.02	-10	(-20,1)	0.06	-8	(-16,1)	0.08
ALT[4]												
3 y	3	(-3,10)	0.28	9	(6,13)	<0.01	3	(-3,9)	0.36	8	(5, 11)	<0.01
3-7 y	43	(10,86)	0.01	38	(25, 52)	<0.01	45	(10,92)	0.01	33	(21, 47)	<0.01
7-13 y	-17	(-37,8)	0.16	-21	(-32,-8)	<0.01	-18	(-37,8)	0.17	-18	(-29,-5)	0.01
AST[4]												
3 y	1	(-2,3)	0.63	3	(1,5)	<0.01	0	(-2,3)	0.75	3	(1,4)	0.01
3-7 y	15	(3,28)	0.01	13	(6, 21)	<0.01	16	(4,30)	0.01	12	(5, 19)	<0.01
7-13 y	-2	(0.89,9)	0.75	-5	(-14,4)	0.27	-1	(-11,10)	0.80	-4	(-13,5)	0.35
GGT[4]												
3 y	5	(2,9)	<0.01	6	(3,9)	<0.01	5	(2,9)	<0.01	5	(3,8)	<0.01
3-7 y	4	(-8,19)	0.52	20	(11, 31)	<0.01	5	(-9,20)	0.53	17	(8, 27)	<0.01
7-13 y	0	(-12,15)	0.96	-13	(-24,-1)	0.04	0	(-13,15)	0.98	-12	(-22,0)	0.05

[1]Coefficients for per 100 kcal increase in energy intake at age 3 or per 100 kcal/year increase in energy intake from ages 3-7 and 7-13 y
[2] adjusted for sex, age at outcome assessment, maternal pre-pregnancy BMI, maternal age, social class, maternal education and parity.
[3] N=1992 for USS liver outcomes at all ages
[4] N=2338 for blood-based liver outcomes at all ages
ALT – alanine aminotransferase; AST – aspartate aminotransferase; GGT- gamma glutamyl transferase; USS – ultrasound scan

Online Supporting Material

Supplemental Table 12: Associations of energy intake at ages 3, 7 and 13 y with USS liver stiffness (n=1992) and blood-based (n=2338) liver outcomes at mean age 17.8 y with additional adjustment for AUDIT scores

	Unadjusted			Adjusted for potential confounders[2]			Adjusted for potential confounders and mediators[3]		
	Energy intake								
	OR[1]	95% CI	p	OR[1]	95% CI	p	OR[1]	95% CI	p
USS-measured liver fat[4]									
3 y	1.77	(1.23,2.57)	<0.01	1.79	(1.14,2.79)	0.01	1.17	(0.68, 2.00)	0.57
7 y	1.39	(1.15,1.67)	<0.01	1.30	(1.06,1.60)	0.01	1.05	(0.82, 1.36)	0.70
13 y	1.07	(0.91,1.26)	0.38	1.11	(0.83,1.49)	0.47	0.81	(0.58, 1.13)	0.21
	% Change[1]	95% CI	p	% Change[1]	95% CI	p	% Change[1]	95% CI	p
USS liver stiffness[4]									
3 y	1	(0,3)	0.04	1	(0,3)	0.16	0	(-1, 2)	0.62
7 y	1	(0,2)	<0.01	1	(0,1)	0.07	0	(0, 1)	0.35
13 y	0	(0,1)	0.39	0	(-1,1)	0.49	0	(-1, 1)	0.75
ALT[5]									
3 y	12	(10,15)	<0.01	7	(4,10)	<0.01	3	(0, 6)	0.04
7 y	6	(4,7)	<0.01	3	(2,5)	<0.01	1	(0, 3)	0.03
13 y	5	(4,6)	<0.01	4	(2,5)	<0.01	2	(0, 4)	0.02
AST[5]									
3 y	7	(6,9)	<0.01	1	(-1,3)	0.27	1	(-1, 2)	0.59
7 y	3	(2,3)	<0.01	1	(0,1)	0.10	0	(0, 1)	0.27
13 y	4	(3,4)	<0.01	1	(0,2)	0.04	1	(0, 2)	0.09
GGT[5]									
3 y	12	(10,14)	<0.01	4	(2,7)	<0.01	0	(-2, 3)	0.73
7 y	5	(4,6)	<0.01	2	(1,3)	<0.01	0	(-1, 1)	0.86
13 y	6	(5,7)	<0.01	2	(1,4)	<0.01	0	(-1, 2)	0.55

[1]Coefficients for energy intake are per 100 kcal increase in energy intake

[2] adjusted for sex, age at outcome assessment, maternal pre-pregnancy BMI, maternal age, social class, maternal education and parity.

[3] additionally adjusted for total body fatness at the time of outcome assessment

[4] N=1992 for USS liver outcomes at all ages

[5] N=2338 for blood-based liver outcomes at all ages

ALT – alanine aminotransferase; AST – aspartate aminotransferase; GGT- gamma glutamyl transferase; USS – ultrasound scan

Online Supporting Material

Supplemental Table 13: Associations of energy intake at age 13 y with USS liver stiffness (n=1992) and blood-based (n=2338) liver outcomes at mean age 17.8 y with additional adjustment for physical activity and puberty

	Unadjusted			Adjusted for potential confounders[2]			Adjusted for potential confounders and mediators[3]		
	Energy intake								
	OR[1]	95% CI	p	OR[1]	95% CI	p	OR[1]	95% CI	p
USS-measured liver fat[4]									
13 y	1.07	(0.91,1.26)	0.38	1.23	(0.88,1.72)	0.23	0.84	(0.58, 1.22)	0.36
	% Change[1]	95% CI	p	% Change[1]	95% CI	p	% Change[1]	95% CI	p
USS liver stiffness[4]									
13 y	0	(0,1)	0.39	1	(0,2)	0.13	0	(-1, 1)	0.56
ALT[5]									
13 y	5	(4,6)	<0.01	4	(2,5)	<0.01	2	(0,3)	0.05
AST[5]									
13 y	4	(3,4)	<0.01	1	(0,2)	0.02	1	(0,2)	0.05
GGT[5]									
13 y	6	(5,7)	<0.01	2	(1,3)	<0.01	0	(-1,1)	-3

[1]Coefficients for energy intake are per 100 kcal increase in energy intake
[2]adjusted for sex, age at outcome assessment, maternal pre-pregnancy BMI, maternal age, social class, maternal education and parity.
[3] additionally adjusted for total body fatness at the time of outcome assessment
[4] N=1992 for USS liver outcomes at all ages
[5] N=2338 for blood-based liver outcomes at all ages
ALT – alanine aminotransferase; AST – aspartate aminotransferase; GGT- gamma glutamyl transferase; USS – ultrasound scan

Online Supporting Material

Supplemental References

1. Schwimmer JB, Deutsch R, Kahen T, Lavine JE, Stanley C, Behling C. Prevalence of Fatty Liver in Children and Adolescents. Pediatrics. 2006 Oct;118:1388-93.

2. Debbie A Lawlor, Mark Callaway, Corrie Macdonald-Wallis, Emma Anderson, Abi Fraser, Laura D Howe, Chris Day, Naveed Sattar. Non-alcoholic fatty liver disease, liver fibrosis and cardiometabolic risk factors in adolescence: a population study of 1874 adolescents. Journal of Clinical Endocrinology & Metabolism. 2014 Mar;99(3):E410-E417.

3. Dowman JK, Tomlinson JW, Newsome PN. Pathogenesis of non-alcoholic fatty liver disease. QJM. 2010 Feb;103:71-83.

4. Donnelly KL, Smith CI, Schwarzenberg SJ, Jessurun J, Boldt MD, Parks EJ. Sources of fatty acids stored in liver and secreted via lipoproteins in patients with nonalcoholic fatty liver disease. J Clin Invest. 2005 May;115:1343-51.

5. Appel LJ, Moore TJ, Obarzanek E, Vollmer WM, Svetkey LP, Sacks FM, Bray GA, Vogt TM, Cutler JA, et al. A Clinical Trial of the Effects of Dietary Patterns on Blood Pressure. N Engl J Med. 1997 Apr 17;336:1117-24.

6. Zhao G, Etherton TD, Martin KR, West SG, Gillies PJ, Kris-Etherton PM. Dietary Linolenic Acid Reduces Inflammatory and Lipid Cardiovascular Risk Factors in Hypercholesterolemic Men and Women. The Journal of Nutrition. 2004 Nov 1;134:2991-7.

7. Stern L, Iqbal N, Seshadri P, Chicano KL, Daily DA, McGrory J, Williams M, Gracely EJ, Samaha FF. The Effects of Low-Carbohydrate versus Conventional Weight Loss Diets in Severely Obese Adults: One-Year Follow-up of a Randomized Trial. Annals of Internal Medicine. 2004 May 18;140:778-85.

8. Mager DR, Patterson C, So S, Rogenstein CD, Wykes LJ, Roberts EA. Dietary and physical activity patterns in children with fatty liver. Eur J Clin Nutr. 2010 Jun;64:628-35.

9. Papandreou D, Rousso I, Malindretos P, Makedou A, Moudiou T, Pidonia I, Pantoleon A, Economou I, Mavromichalis I. Are saturated fatty acids and insulin resistance associated with fatty liver in obese children? Clin Nutr. 2008 Apr;27:233-40.

10. Papandreou D, Karabouta Z, Pantoleon A, Rousso I. Investigation of anthropometric, biochemical and dietary parameters of obese children with and without non-alcoholic fatty liver disease. Appetite. 2012 Dec;59:939-44.

11. Quiros-Tejeira RE, Rivera CA, Ziba TT, Mehta N, Smith CW, Butte NF. Risk for nonalcoholic fatty liver disease in Hispanic youth with BMI > or =95th percentile. J Pediatr Gastroenterol Nutr. 2007 Feb;44:228-36.

12. Vos MB, Colvin R, Belt P, Molleston JP, Murray KF, Rosenthal P, Schwimmer JB, Tonascia J, Unalp A, Lavine JE. Correlation of vitamin E, uric acid, and diet composition

with histologic features of pediatric NAFLD. J Pediatr Gastroenterol Nutr. 2012 Jan;54:90-6.

13. Vos MB, Colvin R, Belt P, Molleston JP, Murray KF, Rosenthal P, Schwimmer JB, Tonascia J, Unalp A, Lavine JE. Correlation of vitamin E, uric acid, and diet composition with histologic features of pediatric NAFLD. J Pediatr Gastroenterol Nutr. 2012 Jan;54:90-6.

14. Oddy WH, Herbison CE, Jacoby P, Ambrosini GL, O'Sullivan TA, Ayonrinde OT, Olynyk JK, Black LJ, Beilin LJ, et al. The Western dietary pattern is prospectively associated with nonalcoholic fatty liver disease in adolescence. Am J Gastroenterol. 2013 May;108:778-85.

15. Boyd A, Golding J, Macleod J, Lawlor DA, Fraser A, Henderson J, Molloy L, Ness A, Ring S, Davey Smith G. Cohort Profile: The 'Children of the 90s' - the index offspring of the Avon Longitudinal Study of Parents and Children. International Journal of Epidemiology. 2012 Apr 16;42:111-27.

16. Fraser A, Macdonald-Wallis C, Tilling K, Boyd A, Golding J, Davey Smith G, Henderson J, Macleod J, Molloy L, et al. Cohort Profile: The Avon Longitudinal Study of Parents and Children: ALSPAC mothers cohort. International Journal of Epidemiology. 2012 Apr 16;42:97-110.

17. Roldan-Valadez E, Favila R, Martinez-Lopez M, Uribe M, Mendez-Sanchez N. Imaging techniques for assessing hepatic fat content in nonalcoholic fatty liver disease. Ann Hepatol. 2008 Jul;7:212-20.

18. Noruegas MJ, Matos H, Goncalves I, Cipriano MA, Sanches C. Acoustic radiation force impulse-imaging in the assessment of liver fibrosis in children. Pediatr Radiol. 2012 Feb;42:201-4.

19. Emmett P. Dietary assessment in the Avon Longitudinal Study of Parents and Children. Eur J Clin Nutr. 2009 Feb;63 Suppl 1:S38-S44.

20. Saunders JB, Aasland OG, Babor TF, de la Fuente JR, Grant M. Development of the Alcohol Use Disorders Identification Test (AUDIT): WHO Collaborative Project on Early Detection of Persons with Harmful Alcohol Consumption--II. Addiction. 1993 Jun;88:791-804.

21. Anderson EL, Tilling K, Fraser A, Macdonald-Wallis C, Emmett P, Cribb V, Northstone K, Lawlor DA, Howe LD. Estimating trajectories of energy intake through childhood and adolescence using linear-spline multilevel models. Epidemiology. 2013 Jul;24:507-15.

22. Rasbash J, Charlton C, Browne WJ, Healy M, Cameron B. MLwiN Version 2.02. Centre for Multilevel Modelling, University of Bristol; 2005.

23. Leckie G, Charlton C. runmlwin: Stata module for fitting multilevel models in the MLwiN software package.Centre for Multilevel Modelling, University of Bristol. 2011.

24. Willet WC. Nutritional Epidemiology. 2nd ed. New York: Oxford University press; 1989.

25. Hattar LN, Wilson TA, Tabotabo LA, Smith EO, Abrams SH. Physical activity and nutrition attitudes in obese Hispanic children with non-alcoholic steatohepatitis. World J Gastroenterol. 2011 Oct 21;17:4396-403.

26. Papandreou D, Rousso I, Malindretos P, Makedou A, Moudiou T, Pidonia I, Pantoleon A, Economou I, Mavromichalis I. Are saturated fatty acids and insulin resistance associated with fatty liver in obese children? Clinical nutrition (Edinburgh, Scotland). 2008;2008/02/01:233-40.

27. Hernaez R, Lazo M, Bonekamp S, Kamel I, Brancati FL, Guallar E, Clark JM. Diagnostic accuracy and reliability of ultrasonography for the detection of fatty liver: a meta-analysis. Hepatology. 2011 Sep 2;54:1082-90.

28. Cole SR, Platt RW, Schisterman EF, Chu H, Westreich D, Richardson D, Poole C. Illustrating bias due to conditioning on a collider. International Journal of Epidemiology. 2010 Apr 1;39:417-20.

29. Liu W, Brookhart MA, Schneeweiss S, Mi X, Setoguchi S. Implications of M Bias in Epidemiologic Studies: A Simulation Study. Am J Epidemiol. 2012 Nov 15;176:938-48.

30. Rogers I, Emmett P. Diet during pregnancy in a population of pregnant women in South West England. ALSPAC Study Team. Avon Longitudinal Study of Pregnancy and Childhood. Eur J Clin Nutr. 1998 Apr;52:246-50.

31. Price GM, Paul AA, Key FB, Harter AC, Cole TJ, Day KC, Wadsworth MEJ. Measurement of diet in a large national survey: comparison of computerized and manual coding of records in household measures. Journal of Human Nutrition and Dietetics. 1995 Dec 1;8:417-28.

32. Wrieden WL, Longbottom PJ, Adamson AJ, Ogston SA, Payne A, Haleem MA, Barton KL. Estimation of typical food portion sizes for children of different ages in Great Britain. Br J Nutr. 2008 Jun;99:1344-53.

33. Gregory J, Lowe S, Bates CJ, Prentice A, Jackson LV, Smithers G, Wenlock R, Farron M. National Diet and Nutrition Survey: young people aged 4 to 18y. Volume I: Report of the diet and nutrition survey.London: The Stationery Office; 2000.

34. Gregory JR, Collins DL, Davies PSW, Hughes JM, Clarke PC. The Diet and Nutrition Survey: children aged 1½–4½y. Vol. 1, Report of the diet and nutrition survey.London: The Stationery Office; 1995.

35. Ministry of Agriculture Fisheries and Food. Food Portion Sizes. 2nd edition ed. London: The Stationery Office; 1993.

36. Marshall WA, Tanner JM. Variations in the Pattern of Pubertal Changes in Boys. Archives of Disease in Childhood. 1970 Feb 1;45:13-23.

37. Marshall WA, Tanner JM. Variations in pattern of pubertal changes in girls. Arch Dis Child. 1969 Jun;44:291-303.

38. Actigraph activity monitors LLC [Internet]. Fort Walton Beach, Florida [date unknown]:[cited 2015 Feb 20]. Available http://www.actigraphcorp.com/product-category/activity-monitors.

39. Royston P. Multiple imputation of missing values. Stata J. 2004;4:227-41.
